# The redox state of the apoplast influences the acclimation of photosynthesis and leaf metabolism to changing irradiance

**DOI:** 10.1111/pce.12960

**Published:** 2017-05-23

**Authors:** Barbara Karpinska, Kaiming Zhang, Brwa Rasool, Daria Pastok, Jenny Morris, Susan R. Verrall, Pete E. Hedley, Robert D. Hancock, Christine H. Foyer

**Affiliations:** ^1^ Centre for Plant Sciences, School of Biology, Faculty of Biological Sciences University of Leeds Leeds LS2 9JT UK; ^2^ College of Forestry Henan Agricultural University Zhengzhou 450002 China; ^3^ Cell and Molecular Sciences The James Hutton Institute Invergowrie Dundee DD2 5DA UK; ^4^ Information and Computational Sciences The James Hutton Institute Invergowrie Dundee DD2 5DA UK

**Keywords:** ascorbate, light acclimation, photosynthesis, redox regulation, transcriptome

## Abstract

The redox state of the apoplast is largely determined by ascorbate oxidase (AO) activity. The influence of AO activity on leaf acclimation to changing irradiance was explored in wild‐type (WT) and transgenic tobacco (*Nicotiana tobaccum*) lines containing either high [pumpkin AO (PAO)] or low [tobacco AO (TAO)] AO activity at low [low light (LL); 250 *μ*mol m^−2^ s^−1^] and high [high light (HL); 1600 *μ*mol m^−2^ s^−1^] irradiance and following the transition from HL to LL. AO activities changed over the photoperiod, particularly in the PAO plants. AO activity had little effect on leaf ascorbate, which was significantly higher under HL than under LL. Apoplastic ascorbate/dehydroascorbate (DHA) ratios and threonate levels were modified by AO activity. Despite decreased levels of transcripts encoding ascorbate synthesis enzymes, leaf ascorbate increased over the first photoperiod following the transition from HL to LL, to much higher levels than LL‐grown plants. Photosynthesis rates were significantly higher in the TAO leaves than in WT or PAO plants grown under HL but not under LL. Sub‐sets of amino acids and fatty acids were lower in TAO and WT leaves than in the PAO plants under HL, and following the transition to LL. Light acclimation processes are therefore influenced by the apoplastic as well as chloroplastic redox state.

## Abstract 2

Understanding the mechanisms that contribute to stress tolerance is vital to future crop productivity. Here, we present data showing that the acclimation of tobacco leaves to high light is influenced by apoplastic ascorbate/dehydroascorbate (DHA) ratios, that is, the redox state of the apoplast. Photosynthesis rates were significantly higher, and the levels of sub‐sets of amino acids and fatty acids were lower in high‐light‐grown leaves when the apoplast was less oxidized because of low ascorbate oxidase activity. These findings demonstrate that the redox state of the apoplast determines the extent of susceptibility of photosynthesis to high‐light‐induced inhibition.

## Introduction

Environmental abiotic stresses such as heat and drought have adverse effects on plant growth, reducing yield in the field. The negative impact of these stresses on crop yields is predicted to increase in coming decades as a result of global climate change. A much deeper understanding of the signalling/response networks that plants use to perceive and acclimate to changing environmental conditions is required for the precise selection of molecular markers and traits underpinning climate‐resilient crop plants. Plants meticulously sense a wide range of external triggers and metabolic cues in order to achieve precise and appropriate acclimation to prevailing environmental conditions (Foyer *et al*. 2016). Perception of environmental changes involves both local intracellular signalling networks in the cells perceiving the stress signal and systemic long‐distance extracellular communication pathways that confer a pre‐emptive general response throughout the plant to immediate and future challenges (Foyer *et al*. 2016). Moreover, perception of one type of stress can trigger enhanced tolerance to a wide range of stresses (Foyer *et al*. 2016), enabling plants to achieve a broad, high level of tolerance to a wide range of potential threats and so improve overall survival and yield under stress.

Two major types of systemic responses have been described in plants: systemic acquired resistance that is typically triggered by pathogens, insect herbivores and physical injury and systemic acquired acclimation (SAA) that is induced by abiotic stress stimuli but particularly high light (HL). Both types of response involve enhanced accumulation of reactive oxygen species (ROS) and oxidative signalling that propagates systemically as a wave, usually as a result of activation of NADPH oxidases (Gilroy *et al*. 2014). Systemic ROS signalling activates many ROS‐responsive transcripts, including the core environmental stress response genes ensuring coordinated and synchronized responses at the whole‐plant level.

Environmental signals are perceived at the external surface of the plasma membrane of individual cells by receptor‐like proteins, particularly receptor‐like protein kinases (RLKs) (Foyer *et al*. 2016). These proteins, which relay information from the extracellular environment to the intracellular compartments, are responsive to ROS production and the redox state of the apoplast. However, many facets of the local and systemic reduction/oxidation (redox) signalling networks that integrate ROS‐dependent and ROS‐independent RLK signalling remain unclear. The central hypothesis guiding the studies described here is that the redox state of the apoplast leading to activation/deactivation of protein sensors participates in the acclimation of leaves to light signals. This hypothesis was tested by altering the redox state of the apoplast by manipulation of the apoplastic enzyme ascorbate oxidase (AO), which catalyses the first step in the degradation of the major low‐molecular‐weight antioxidant, ascorbic acid (Green and Fry 2005, De Tullio *et al*. 2013).

The ability to acclimate to sun and shade environments, as well as changes in irradiance, is key to the regulation of photosynthesis. It is often assumed that the light acclimation process is controlled by signals arising from the chloroplast, but oxidative signals generated in the apoplast also exert an influence over the acclimation process (Guo *et al*. 2016). Light is also an important regulator of ascorbate biosynthesis (Yabuta *et al*. 2007). Sun‐exposed leaves contain substantially more ascorbate than shade leaves (Grace and Logan 1996, Smirnoff and Pallanca 1996). The quantity and quality of light experienced during the photoperiod also exert a strong influence on the extent of leaf ascorbate accumulation (Bartoli *et al*. 2009). Much less is known about the regulation of ascorbate degradation than synthesis. AO belongs to the multicopper oxidase family of enzymes that are classified by their substrate specificity; that is, AOs oxidize ascorbate to dehydroascorbate (DHA) and laccases, which oxidize aromatic substrates such as diphenols. Our previous work has shown that the ascorbate/DHA ratios of the leaf apoplast/cell wall compartment were altered in transformed tobacco lines by expression of either a pumpkin AO (PAO) gene or a tobacco AO (TAO) gene in the antisense orientation without having significant effects on the whole‐leaf ascorbate pool (Pignocchi *et al*. 2003, 2006). Moreover, the AO‐induced changes in ascorbate/DHA ratios of the apoplast altered plant responses to biotic and abiotic stresses (Pignocchi *et al*. 2003, 2006, Sanmartin *et al*. 2003, Yamamoto *et al*. 2005). In the following studies, we used the wild‐type (WT), PAO and TAO tobacco lines to investigate the effects of deregulated changes in AO expression and activity on the acclimation of leaves to changing light levels. The data presented here show that leaf AO activity and the redox state of the apoplast influence the acclimation of leaves to changing light levels.

## Materials and Methods

### Plant material and growth conditions

The generation of transgenic tobacco (Nicotiana tabacum L.; T3 generation) lines expressing a PAO (Cucurbita maxima) gene in the sense orientation (GenBank accession number X55779) or a partial TAO sequence in the antisense orientation (GenBank accession number D43624) has been described previously (Pignocchi *et al*. 2003, 2006). Plants were grown in compost in controlled‐environment chambers at constant relative humidity (60%) and temperature (20 °C) under an 8 h day/16 h night cycle.

All plants were grown for 3 weeks under low light (LL; 250 *μ*mol m^−2^ s^−1^), after which half of the plants were transferred to HL (1600 *μ*mol m^−2^ s^−1^) for a further 7 d, while the other half were maintained under LL conditions. At the end of the light period on the seventh day under contrasting light conditions fully expanded, matched leaf samples were harvested for biochemical measurements, and transcript and metabolite profiling. The remainder of the plant was destroyed. Samples were harvested from individual plants, providing a number of independent biological replicates as described in the figure legends. Plants that had not been previously sampled were maintained in the growth chambers, and at the end of the subsequent dark period, leaf samples were taken again for biochemical measurements as described. All plants were maintained under LL conditions for the next light period, and leaves were harvested from fresh (previously unsampled) plants every 2 h until the end of the light period to provide contrasting samples from leaves that had a history of HL exposure against those that had only been exposed to LL for biochemical measurements. Additional sets of leaf samples were harvested at the end of this photoperiod for transcript and metabolite profiling.

The experiment was repeated at least three times, giving similar results, and data from a representative experiment are shown.

### Shoot phenotype

At the end of the photoperiod on the seventh day, 10 independent 4‐week‐old tobacco plants per genotype and growth irradiance were photographed using a Canon (Tokyo, Japan) EOS 450D digital camera mounted at a set height above the pots. The digital images captured were used to estimate the number of fully expanded leaves, and leaf area was estimated using the image J program version 1.41a scaled to a ruler placed alongside each image.

### Ascorbate oxidase activity

The youngest fully expanded leaves were harvested from three independent 4‐week‐old tobacco plants per genotype per time point as described earlier and immediately frozen in liquid nitrogen. Frozen leaf tissue was ground to a fine powder, 0.1 m sodium phosphate buffer (pH 6.5) was added at a ratio of 10 mL g^−1^ fresh weight (FW) and the mixture was ground until the buffer thawed. The extract was centrifuged for 10 min at 15 000 *g* and 4 °C. The supernatant was discarded, and the pellet was re‐suspended in 0.1 m sodium phosphate (pH 6.5) containing 1 m NaCl. Insoluble material was pelleted again by centrifugation (10 min, 15 000 *g*, 4 °C), leaving proteins ionically bound to the cell wall fraction in the supernatant. Maximal extractable AO activity was estimated at 25 °C as described by Pignocchi *et al*. (2003), via the decrease in absorbance at 265 nm following the addition of 50 *μ*L of extract in a reaction mixture containing 0.1 m sodium phosphate (pH 5.6), 0.5 mm ethylenediaminetetraacetic acid (EDTA) and 100 *μ* m ascorbate. One unit of AO activity is defined as the amount of enzyme required to oxidize 1 *μ*mol of ascorbate per minute.

### Whole‐leaf ascorbate and dehydroascorbate

Whole‐leaf samples from three independent plants per genotype per time point (as indicated on the figures) were harvested, weighed and immediately ground in liquid nitrogen under the prevailing dark/light conditions in the controlled environment chambers. Ascorbate and DHA were extracted from the frozen pellets by grinding again in 1 m HClO_4_ at a ratio of 10 mL g^−1^ FW and assayed as described by Queval and Noctor (2007). Leaf ascorbate and DHA were calculated as described by Noctor *et al*. (2016).

### Apoplastic ascorbate

Apoplastic washing fluid was isolated as described by Pignocchi *et al*. (2003) following vacuum infiltration of young fully expanded leaves at −70 kPa with chilled 10 mm citrate buffer (pH 3.0) for 5 min. Leaves were then blotted dry, carefully rolled and inserted into a pre‐chilled syringe. The apoplastic washing fluid was then collected by placing the syringe into a tube and centrifuging at 2000 g for 10 min at 4 °C. The washing fluid was then diluted into acid, and ascorbate and DHA were measured as for the whole leaf. Data are presented as micromole ascorbate/DHA per gram of FW tissue estimated from the quantity of apoplastic fluid isolated per gram of FW.

### Chlorophyll and carotenoids

Photosynthetic pigments were estimated in the youngest fully expanded leaves harvested from three independent 4‐week‐old plants per genotype per time point. Leaves were weighed and ground in liquid nitrogen and ice‐cold 95% ethanol added at a ratio of 10 mL g^−1^ leaf FW. Extracts were centrifuged for 10 min, at 14 000 *g* (4°C), and the supernatant fractions were used for pigment determination. Chlorophyll and carotenoids were measured by spectrophotometry according to the method of Lichtenthaler (1986).

### Photosynthetic gas exchange

Photosynthetic CO_2_ assimilation, transpiration, stomatal conductance and intracellular CO_2_ were measured on the youngest fully expanded leaves of six independent plants per genotype using a portable infrared gas analyser model LI‐6400XT (Li‐Cor, Lincoln, NE, USA). Measurements were performed at the end of the photoperiod on the seventh day of HL treatment at 20 °C using a light intensity of 250 *μ*mol m^−2^ s^−1^ photosynthetically active radiation (PAR) and an atmospheric CO_2_ concentration of 400 *μ*mol mol^−1^. Leaves were allowed to acclimatize to the chamber for at least 15 min prior to measurement. PAR levels were chosen to eliminate the possibility of HL stress during gas exchange measurements in the LL‐grown plants.

### Microarray processing and analysis

Microarray experiments were conducted to compare gene expression in fully expanded leaves of tobacco genotypes (WT, antisense TAO and sense PAO) grown under LL or HL and harvested either at the end of the final HL period or following 24 h recovery as described under the plant material and growth conditions section. Three independent biological replicates were analysed for each condition and time point, and full experimental design and microarray datasets are available at ArrayExpress (http://www.ebi.ac.uk/arrayexpress), accession E‐MTAB‐4816.

Microarray design ID 021113 (Agilent Technologies, Palo Alto, CA, USA) was used with 43 803 probes representing tobacco transcript sequences. The One‐Color Microarray‐Based Gene Expression Analysis protocol (v. 6.5, Agilent Technologies) was used throughout for microarray processing. Briefly, complementary RNA (cRNA) was synthesized from complementary DNA (cDNA), which was then linearly amplified and labelled with Cy3 prior to purification. Labelled samples were hybridized to microarrays overnight at 65 °C, prior to being washed once for 1 min with GE Wash 1 buffer (Agilent Technologies) at room temperature and once for 1 min with GE Wash Buffer 2 (Agilent Technologies) at 37 °C, and then dried by centrifugation. The hybridized slides were scanned using the Agilent G2505B scanner at a resolution of 5 *μ*m at 532 nm.

feature extraction (FE) software (v. 10.7.3.1, Agilent Technologies) with default settings was used for data extraction from the image files. Subsequent data quality control, pre‐processing and analyses were performed using genespring GX (v. 7.3; Agilent Technologies) software. Agilent FE one‐colour settings in GeneSpring were used to normalize data, and a filter was used to remove inconsistent probe data, flagged as present or marginal in less than 2 out of 18 samples. Two‐way analysis of variance (anova) using the factors light and genotype was used to identify significant differentially expressed probes with a *P*‐value ≤0.05 with Benjamini–Hochberg multiple testing correction. Light‐dependent lists were further trimmed by removing any transcript that exhibited a less than twofold change in abundance between LL and HL conditions in at least one of the tobacco lines.

### Metabolite profiling by gas chromatography/mass spectrometry

Gas chromatography/mass spectrometry (GC/MS) analysis was performed on extracts from three biological replicates per treatment, essentially as described by Foito *et al*. (2013). The youngest fully expanded leaf was snap frozen in liquid nitrogen and then lyophilized. Dried material (100 ± 5 mg) was weighed into glass tubes and extracted in 3 mL methanol for 30 min at 30 °C with agitation (1500 rpm). Polar (ribitol 2 mg mL^−1^) and non‐polar (nonadecanoic acid methyl ester 0.2 mg mL^−1^) standards at 0.1 mL each and 0.75 mL distilled H_2_O were added, and extraction continued for a further 30 min as described. 6 mL of chloroform were added, and extraction continued for 30 min under increased agitation at 2500 rpm. Phase separation was achieved by the addition of a further 1.5 mL of water and centrifugation at 1000 *g* for 10 min. Following oximation, polar metabolites were converted to trimethylsilyl derivatives, while non‐polar metabolites were subjected to methanolysis and trimethylsilylation as described (Foito *et al*. 2013). Metabolite profiles for the polar and non‐polar fractions were acquired following separation of compounds on a DB5‐MSTM column (15 m × 0.25 mm × 0.25 *μ*m; J&W, Folsom, CA, USA) using a Thermo Finnigan (San Jose, CA, USA) DSQII GC/MS system as described (Foito *et al*., 2013). Data were then processed using the xcalibur software (Thermo Fisher Scientific, Waltham, MA, USA). Peak areas relative to internal standard (response ratios) were calculated following normalization to 100 mg extracted material.

### Accession numbers

Experimental design and microarray datasets are available at ArrayExpress (http://www.ebi.ac.uk/arrayexpress), accession E‐MTAB‐4816.

### Statistical analysis

With the exception of microarray data, which were analysed as described using genespring GX (v. 7.3, Agilent Technologies), all statistical analyses were undertaken by two‐way anova with the factors genotype and light using genstat (v. 18.1, VSN International Ltd, Hemel Hempstead, UK).

## Results

**Figure 1:**
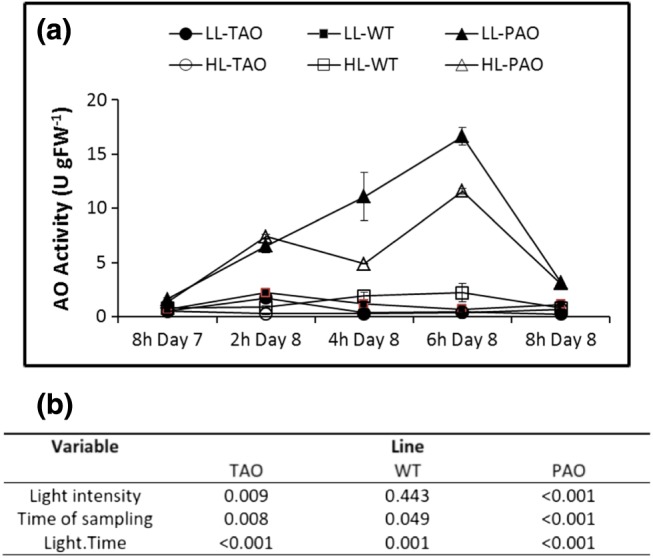
Ascorbate oxidase activity in wild‐type (WT), pumpkin ascorbate oxidase (PAO) and tobacco ascorbate oxidase (TAO) tobacco lines at the end of high‐light (HL) treatment (8 h day 7) or following return to low light (LL) (2 h day 8/8 h day 8); FW, fresh weight (a). Plants were grown for 3 weeks under LL (250 *μ*mol m^−2^ s^−1^) and then maintained for a further 7 d under LL or transferred to HL (1600 *μ*mol m^−2^ s^−1^). At the end of the photoperiod on the seventh day of HL treatment, all plants were returned to an LL regime. Plants were sampled immediately after the end of the final 8 h photoperiod following HL treatment (8 h day 7) or after 2, 4, 6 or 8 h following return to LL. Ascorbate oxidase (AO) activity, defined as the amount of enzyme required to oxidize 1 *μ*mol of ascorbate per minute, was estimated in four biological replicates of antisense TAO, WT and sense PAO plants grown under LL or HL and is represented as mean ± SE. *P*‐values as determined by two‐way analysis of variance using the variables light intensity and time of sampling as well as their interactive effects are provided for each line (b). [Colour figure can be viewed at http://wileyonlinelibrary.com]

Wild‐type and transgenic TAO and PAO tobacco plants were grown for 3 weeks under controlled environment LL conditions (250 *μ*mol m^−2^ s^−1^). Half of the plants were then transferred to HL (1600 *μ*mol m^−2^ s^−1^) conditions for 7 d. Photosynthesis was determined, and leaf samples were harvested for metabolite and transcriptome profiling and for biochemical analysis from plants grown under HL or LL conditions. Following a further dark period, the HL‐grown plants were returned to LL conditions and samples taken every 2 h during the entirety of the 8 h light period. Maximal extractable AO activity was up to almost 25‐fold higher in the leaves of PAO plants than in the WT (Fig. 1). Conversely, the antisense TAO lines had as little as 15% of the maximal extractable AO activity of WT plants. Both light intensity and time under light had a significant effect on maximal extractable AO activities of PAO and TAO leaves (Fig. 1). In particular, the maximal extractable AO activity varied markedly during the photoperiod in the PAO lines, the activities being highest 6 h into the photoperiod and then declining again by the end of the photoperiod (Fig. 1). At all times sampled, the AO activity of PAO lines was significantly (*P* < 0.001) higher than that of WT lines, while TAO lines exhibited a lower AO activity than did WT.

**Figure 2:**
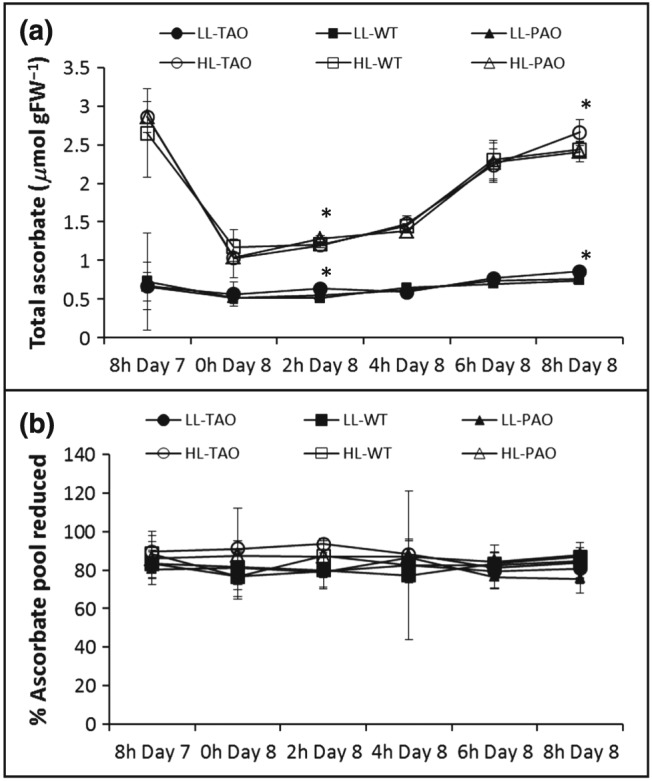
Whole‐leaf ascorbate content of wild‐type (WT), pumpkin ascorbate oxidase (PAO) and tobacco ascorbate oxidase (TAO) tobacco lines at the end of high‐light (HL) treatment or following return to low light (LL). Plants were grown for 3 weeks under LL (250 *μ*mol m^−2^ s^−1^) and then maintained for a further 7 d under LL or transferred to HL (1600 *μ*mol m^−2^ s^−1^). At the end of the photoperiod on the seventh day of HL treatment, all plants were returned to an LL regime. Plants were sampled immediately after the end of the final 8 h photoperiod following HL treatment (8 h day7) or 2, 4, 6 or 8 h into the photoperiod following return to LL. Total ascorbate content (a) and the proportion of the ascorbate pool in the reduced form (b) are indicated as mean ± SE, *n* = 4 or 5. Asterisks indicate significant differences between lines grown in HL or LL (*P* < 0.05).

In agreement with previous findings (Pignocchi *et al*. 2003), changes in AO activities in PAO and TAO leaves had little impact on whole‐leaf ascorbate or ascorbate/DHA ratios (Fig. 2). However, there was a strong and persistent effect of the HL history of the plants on leaf ascorbate content in all lines (Fig. 2). Not only did plants grown under HL for 7 d have a higher leaf ascorbate content at the end of the final HL photoperiod (8 h Day 7), but leaf ascorbate also accumulated to much higher levels than those measured in the LL plants in the subsequent period even though the plants had been exposed to LL conditions throughout the photoperiod (Fig. 2). The HL‐grown plants therefore retained a molecular memory that led to much higher leaf ascorbate following the transition to LL than were observed in leaves that had been grown continuously under LL. Leaf ascorbate contents increased throughout the photoperiod in all genotypes, values being highest at the end of the 8 h photoperiod (Fig. 2). The time into the photoperiod effect on the extent of ascorbate accumulation was also observed in LL‐grown plants.

**Figure 3:**
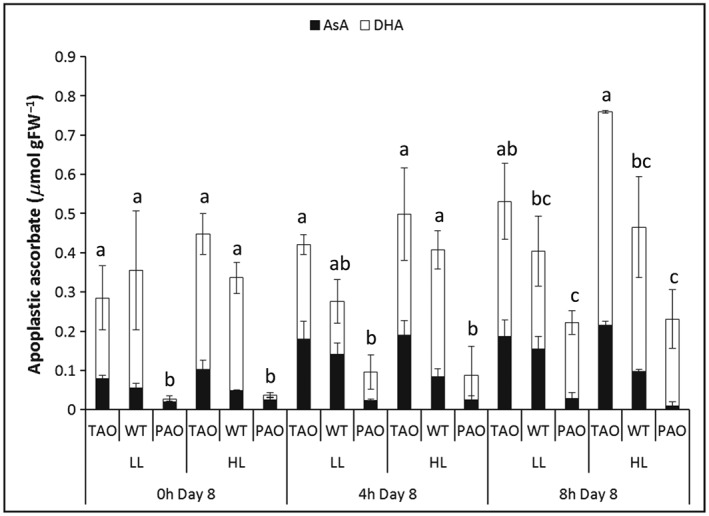
Apoplastic ascorbate (AsA) content of wild‐type (WT), pumpkin AsA oxidase (PAO) and tobacco AsA oxidase (TAO) tobacco lines following return to low light (LL) after 7 d exposure to high light (HL) (1600 *μ*mol m^−2^ s^−1^) or maintenance under LL (250 *μ*mol m^−2^ s^−1^). Plants were harvested at the times indicated and AsA and dehydroascorbate (DHA) estimated as described in materials and methods. Bars represent mean values ± SE, *n* = 3, and samples harvested at the same point with common letters did not exhibit significantly different total apoplastic AsA content as estimated using Fisher's protected least significant difference (LSD) (*P* < 0.05). FW, fresh weight.

In contrast to the total leaf ascorbate pool, AO activity in the TAO and PAO lines resulted in significant differences in the total amount of ascorbate in the leaf apoplast/cell wall compartment (Fig. 3). Furthermore, there was a trend towards greater oxidation of the ascorbate pool as apoplastic AO activity in the different lines increased. On the contrary, previous exposure to HL had little influence on apoplastic ascorbate. The total amount of ascorbate in the leaf apoplast/cell wall compartment increased from the beginning to the end of the photoperiod. This light‐dependent increase in apoplastic ascorbate was greatest in the PAO lines, the levels being 10‐fold higher at the end of the photoperiod than at the beginning, compared to an approximate doubling in apoplastic ascorbate in the TAO lines over the same time period (Fig. 3). The ascorbate/DHA ratios in the leaf apoplast/cell wall compartment also changed during the photoperiod being most oxidized in the PAO lines at the end of the light period (Fig. 3).

### Shoot phenotype is more dependent on growth irradiance than on ascorbate oxidase activity

**Figure 4:**
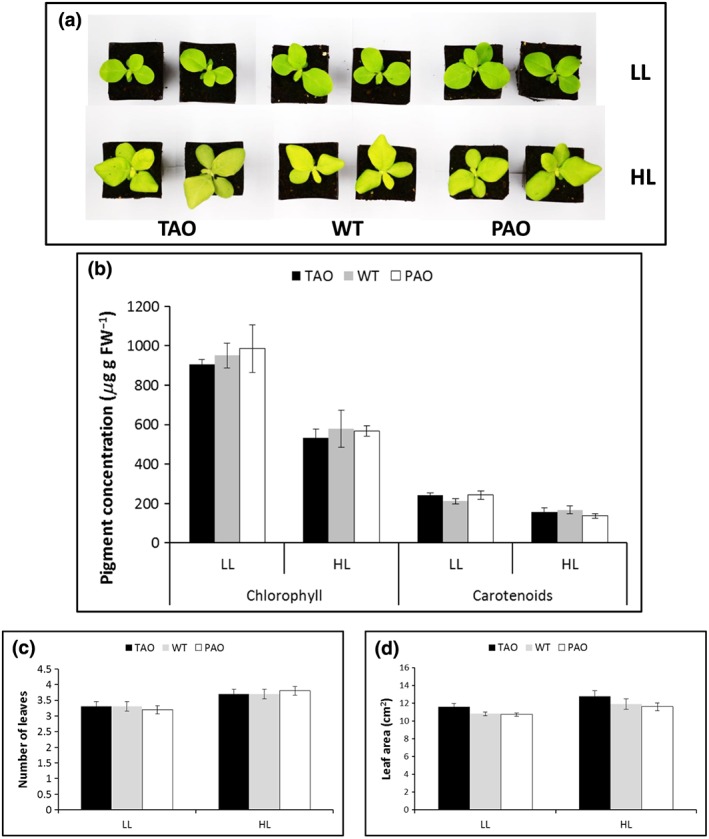
Shoot phenotypes of the wild type (WT), pumpkin ascorbate oxidase (PAO) and tobacco ascorbate oxidase (TAO) tobacco lines. Tobacco plants were grown for 3 weeks under low light (LL) (250 *μ*mol m^−2^ s^−1^) and then maintained for a further 7 d under LL (250 *μ*mol m^−2^ s^−1^) or high light (HL) (1600 *μ*mol m^−2^ s^−1^). Representative photographs (a) and photosynthetic pigments (b; mean ± SE, *n* = 3; FW, fresh weight). Total leaf number (c) and leaf area (d) per plant were estimated from 10 replicate plants and are indicated as mean ± SE.

The leaves of all lines grown for 7 d under HL (Fig. 4a) had significantly less total leaf chlorophyll and carotenoid contents than the LL controls, although no differences between the WT, TAO and PAO lines were observed (two‐way anova, *P* < 0.05) (Fig. 4b). Growth under HL resulted in a significant increase (*P* < 0.05) in leaf number and total leaf area in all lines (Fig. 4c,d). There was additionally a trend towards higher leaf area in plants with the lowest AO activity under both HL and LL (Fig. 4d), although the magnitude of this effect was just outside significance (*P* = 0.059).

### Low ascorbate oxidase activity results in higher photosynthetic carbon assimilation rates under high light

**Figure 5:**
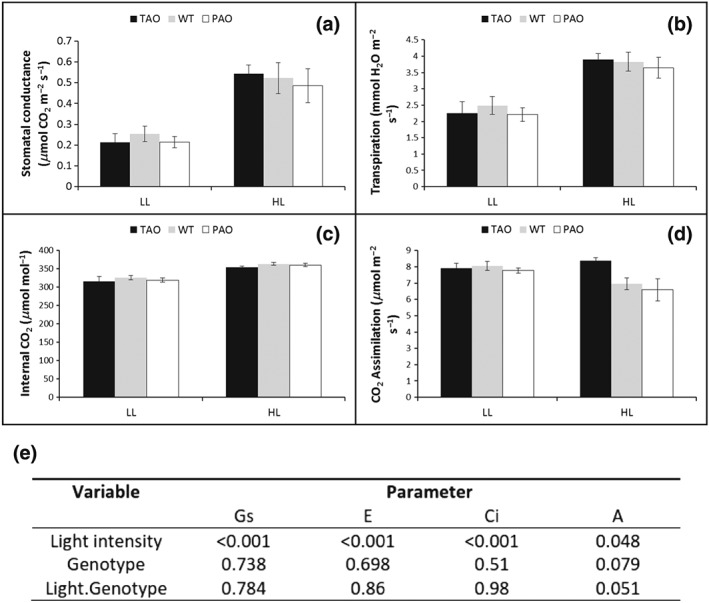
Photosynthetic phenotype of tobacco lines with altered ascorbate oxidase activity grown under low light (LL) or high light (HL) conditions. Tobacco plants were grown for 3 weeks under LL (250 *μ*mol m^−2^ s^−1^) and then maintained for a further 7 d under LL (250 *μ*mol m^−2^ s^−1^) or HL (1600 *μ*mol m^−2^ s^−1^). Measurements were taken on the youngest fully expanded leaves at the end of the photoperiod. (a) Stomatal CO_2_ conductance; (b) transpiration rates; (c) internal CO_2_ concentration and (d) net photosynthesis. All data are presented as mean ± SE, *n* = 4. Significant differences were estimated by two‐way analysis of variance (anova) using the factors light and genotype, and *P*‐values are presented (e). PAO, pumpkin ascorbate oxidase; TAO, tobacco ascorbate oxidase; WT, wild type.

Leaves on plants grown under HL had double the stomatal conductance values of plants grown under LL conditions (Fig. 5a). The increased stomatal conductance rates were associated with large increases in leaf transpiration rates (Fig. 5b) irrespective of leaf AO activity. A small (approximately 10%) but highly significant increase in leaf internal CO_2_ concentration (Ci) was observed in the HL‐grown leaves from all lines (Fig. 4c).

Growth under HL for 7 d decreased the photosynthetic carbon assimilation rates by 10% in the WT and PAO plants. In contrast, photosynthetic carbon assimilation rates in the HL‐grown TAO leaves were similar to those grown under LL (Fig. 5d) as reflected by the fact that the *P*‐value for light by genotype fell just outside the 5% level of significance (Fig. 5e).

### The effect of irradiance on the leaf transcriptome

Leaf transcript profiles were compared at the end of the photoperiod on the seventh day under HL and in plants that had been grown continuously for 4 weeks under LL. For the identification of transcripts differentially abundant as a result of growth irradiance or genotype, two‐way anova (*P* < 0.05) with Benjamini–Hochberg multiple testing correction was applied. This identified 7116 transcripts that changed in abundance in response to growth irradiance. In contrast, using this highly stringent statistical approach, we failed to identify any transcripts whose abundance was dependent on genotype. This surprising result indicates the dominant impact of differences in light irradiance on gene expression profiles relative to the much more subtle influence of plant genotype. In order to further simplify the list of transcripts whose abundance was light dependent, we excluded transcripts that failed to exhibit a minimum of a twofold difference in abundance under HL versus LL in at least one genotype from the analysis. The remaining list of 3859 transcripts (Supporting Information Table S1) were then analysed by Wilcoxon rank sum test using PageMan (Usadel *et al*. 2006). This analysis revealed that several clusters of transcripts associated with specific functions were statistically regulated in abundance by light intensity (Supporting Information Fig. S1). The majority of functional clusters behaved in a similar manner in all genotypes tested. For example, transcripts associated with amino acid degradation and anthocyanin biosynthesis were lower in all genotypes under HL relative to LL (Supporting Information Fig. S1). However, there were also a number of marked differences between the genotypes. Crucially, the PAO plants exhibited a statistically significant increase in abundance of transcripts encoding photosystem II (PSII) polypeptides that was not observed in WT or TAO plants (Supporting Information Fig. S1), suggesting a higher turnover of PSII in plants with low apoplastic redox buffering capacity. In addition, the TAO plants exhibited an enhanced abundance of transcripts associated with aromatic amino acid synthesis and a reduced abundance of transcripts encoding proteinase inhibitors under HL compared to LL (Supporting Information Fig. S1).

### Transcript profiles at the end of the first photoperiod following the transition from high light to low light

Leaf transcript profiles were compared at the end of the first photoperiod following the transition from HL to LL and in plants that had been grown continuously under LL. Following the two‐way anova as described for the data obtained at the end of the HL period, 16 096 transcripts exhibited significant changes in abundance in response to light. This set of transcripts was further reduced as described for the dataset obtained at the end of the HL period by excluding transcripts that failed to exhibit a twofold difference in abundance between LL‐grown and HL‐grown plants in at least one genotype, leaving a total of 4352 transcripts (Supporting Information Table S2). Thus, following recovery under a single LL photoperiod, only approximately 25% of significantly differentially abundant transcripts were more than twofold altered compared with more than 50% immediately after the HL treatment. In contrast, only 14 transcripts were significantly altered in abundance in the different genotypes (Supporting Information Table S2), and a further three transcripts (of which only two mapped onto MapMan) showed changes in abundance that were dependent on an interaction between the light environments and the genotypic background (Supporting Information Table S2). Although numbers of transcripts influenced by genotype were still relatively small, these data indicate that genotypic differences are likely to be most marked when plants are grown in similar environments.

Transcripts significantly differently abundant in response to prior illumination were categorized using the PageMan tool (Supporting Information Fig. S2). This indicated a large increase in abundance in transcripts associated with both PSII and photosystem I (PSI) as may be expected upon transfer from high to low illumination. Transcripts associated with a large number of different processes were also significantly altered in abundance in similar ways in all genotypes. One interesting divergence was the observation that transcripts associated with protein turnover and amino acid synthesis and turnover were significantly more abundant in WT and particularly TAO plants that had a history of HL exposure but that any change was not significant in PAO plants (Supporting Information Fig. S2).

The small numbers of transcripts that exhibited significant differences in abundance between WT, PAO and TAO plants as well as those that exhibited a genotype‐dependent response to light did not exhibit particularly large differences in abundance, with none of them exhibiting a more than twofold change between genotypes (Supporting Information Table S2).

### The transition from high light to low light and ascorbate oxidase activity shape the metabolic profile of tobacco leaves

The leaf metabolite profiles of the TAO, WT and PAO plants were compared in HL and LL (stress) plants and 12 h after the transition from HL to LL (recovery). Metabolites, whose abundance was changed in a statistically significant manner under either stress or recovery, were identified using two‐way anova based on illumination (HL or LL) and genotype. A total of 58 metabolites were significantly influenced by the light environment (Supporting Information Table S3). Fifteen of these exhibited significant changes immediately after the HL stress but were not significantly different following recovery for 12 h under LL. Conversely, the levels of 26 metabolites were not significantly different between LL‐grown and HL‐grown plants but were significantly changed following the transition from HL to LL. A further 17 metabolites were significantly different in HL‐grown and LL‐grown plants and also following the transition from HL to LL.

**Figure 6:**
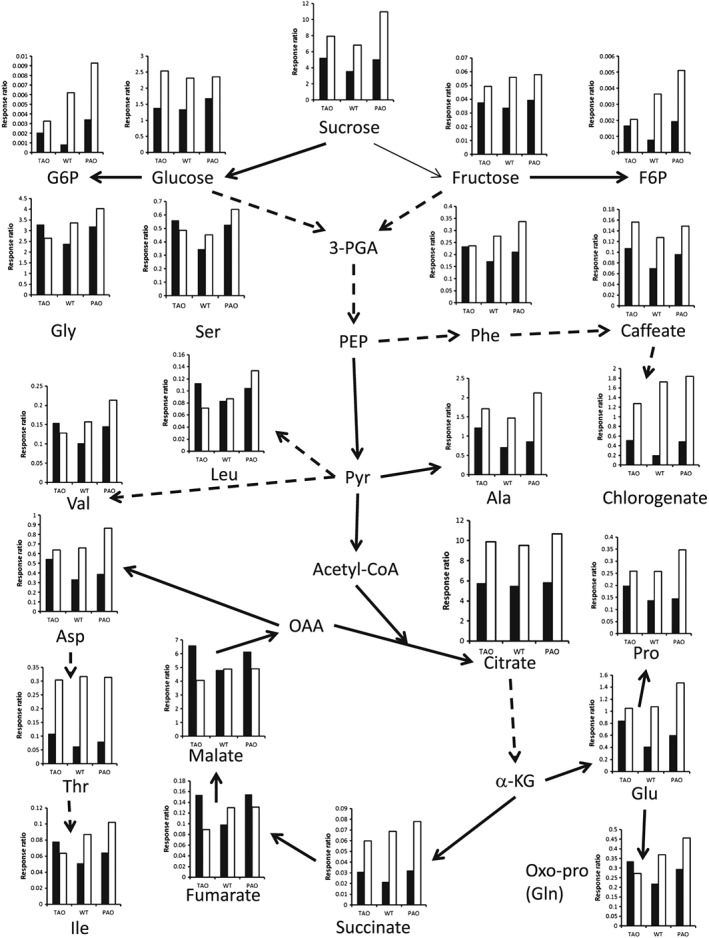
Influence of illumination on selected metabolites in tobacco leaves. TAO, WT and PAO tobacco plants were grown for 3 weeks under LL (250 *μ*mol m^−2^ s^−1^) and then maintained for a further 7 d under LL (250 *μ*mol m^−2^ s^−1^) or HL (1600 *μ*mol m^−2^ s^−1^). Plants were harvested at the end of the photoperiod and immediately frozen in liquid nitrogen prior to processing for GC/MS. Charts illustrate the mean metabolite concentration relative to the internal standard ribitol (response ratio) in TAO, WT and PAO plants grown under LL (black bars) or HL (white bars), *n* = 3. All metabolites illustrated were significantly influenced by light (*P* < 0.05). Arrows indicate metabolic relationships where dashed arrows indicated multiple metabolic steps. 3‐PGA, 3‐phosphoglycerate; CoA, coenzyme A; F6P, fructose 6‐phosphate; G6P, glucose 6‐phosphate; GC/MS, gas chromatography/mass spectrometry; HL, high light; LL, low light; OAA, oxaloacetate; PAO, pumpkin ascorbate oxidase; PEP, phosphoenol pyruvate; TAO, tobacco ascorbate oxidase; WT, wild type; *α*‐KG, *α*‐ketoglutarate. Amino acids are labelled according to the standard three‐letter code.

Significantly higher concentrations of a number of sugars, sugar phosphates and amino acids were found in HL‐grown than in LL‐grown plants (Fig. 6). Citrate and succinate were also significantly higher in HL‐grown plants, while fumarate and malate were on average slightly lower in HL plants (Fig. 6). In addition, caffeic acid and chlorogenic acid were increased 1.6‐fold and 4‐fold, respectively, at HL compared to LL and following the transition from HL to LL (Fig. 6), supporting a role for these phenolic acids as sun screens (Chenier *et al*. 2013). The levels of a subset of fatty acids and the C24 lignoceryl alcohol were significantly decreased following the transfer from HL to LL conditions (Supporting Information Fig. S3). The levels of sugar phosphates were also higher in leaves of plants that had been transferred from HL to LL than those that had been grown continuously under LL (Supporting Information Fig. S3). Similarly, the abundance of certain amino acids, particularly aspartic acid (Asp) and glutamic acid (Glu), was sixfold higher in leaves of plants that had been grown under HL compared to LL (Fig. 6). Moreover, a number of amino acids [threonine (Thr), *β*‐alanine (Ala), Asp, Glu and phenylalanine (Phe)] had a higher abundance both under HL and after the transition from HL to LL, while the levels of several other amino acids were higher only after the transition from HL to LL [valine (Val), leucine (Leu), isoleucine (Ile), glycine (Gly) and serine (Ser)] (Supporting Information Fig. S3). In particular, the levels of Gly were 18‐fold higher in leaves following the transition from HL to LL than leaves grown under LL. The transition from HL to LL also had a significant impact on the levels of a large number of fatty acids, fatty alcohols and phytosterols (Supporting Information Fig. S3). For example, the levels of many saturated and unsaturated medium‐chain fatty acids, including linoleic and linolenic acids, phytol A and many medium–long‐chain (C22–C30) fatty alcohols, stigmasterol and *β*‐sitosterol were significantly lower in leaves following transfer from HL to LL.

**Figure 7:**
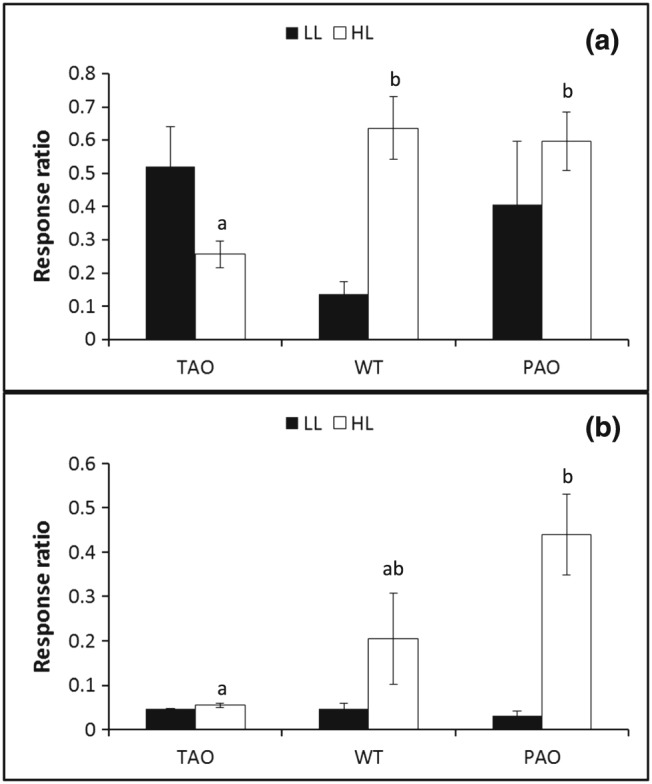
Leaf threonate content of tobacco ascorbate oxidase (TAO), wild‐type (WT) and pumpkin ascorbate oxidase (PAO) tobacco leaves under differing illumination. Leaves were sampled immediately after the end of the light period on the seventh day of high‐light (HL) treatment (a) or following recovery under low light (LL) for a further 12 h (b). Threonate content relative to that of the internal standard ribitol (response ratio) was estimated in the polar fraction by gas chromatography/mass spectrometry (GC/MS) following derivatization, and the graphs indicate mean values ± SE, *n* = 3. Columns labelled with different letters indicate significantly different values (*P* < 0.05) as estimated by Fisher's protected least significant difference (LSD) test.

Alterations in AO activity had a less profound impact on the tobacco leaf metabolome than changes in light intensity. Nevertheless, modifications in AO activity had significant effects on the leaf metabolite profile (Supporting Information Fig. S4). In total, the levels of 20 metabolites were influenced by changes in leaf AO activity (Supporting Information Fig. S4 and Table S3). There was little overlap between metabolites that were altered in relation to AO activity in leaves from HL‐grown and LL‐grown plants and those identified as changed in relation to genotype following the transition from HL to LL. These findings suggest that the AO‐dependent changes in the leaf metabolite profile were strongly influenced by changes in the light environment. Although the levels of a small number of metabolites were changed in an inverse manner in the PAO and TAO plants relative to WT, some metabolites were either more or less abundant in both low‐AO and high‐AO plants compared to the WT (Supporting Information Fig. S4). This may suggest an altered regulation of metabolism resulting from altered levels of AO activity. The levels of threonate, a breakdown product of ascorbate, were lower in TAO leaves than in the WT, while they were increased in the leaves of PAO plants (Fig. 7). This finding confirms the role of AO in ascorbate degradation, particularly following the transition from HL to LL conditions.

Twenty‐nine metabolites were changed in abundance in a manner that showed a genotype × light interaction. For example, fatty acids were significantly lower in WT and TAO leaves following the transition from HL to LL than in those that had been grown under LL conditions. The same fatty acids were also more abundant in PAO leaves (Supporting Information Fig. S5). Changes in AO activity also had a major influence on the levels of *β*‐Ala, Asp and Thr following the transition from HL to LL, where the levels of these amino acids in PAO plants were much higher following the transition from HL to LL than those in plants that had been grown under LL.

## Discussion

Leaves growing in sun and shade environments within plant canopies show structural, developmental and metabolic adjustments in photosynthesis and leaf metabolism, responding to prevailing light availability (Green *et al*. 2003, Leakey *et al*. 2005, Walters 2005, Athanasiou *et al*. 2010, Cohu *et al*. 2014, Demmig‐Adams *et al*. 2014, Muller *et al*. 2014). Exposure to full sunlight means that leaves require much less chlorophyll to maintain high photosynthesis rates than those grown in the shade or under LL (Walters and Horton 1995a,b, Yin and Johnson 2000). The acclimation of photosynthesis and changes in leaf pigments determined in the tobacco leaves studied in the present experiments are entirely consistent with these concepts, as are the light‐dependent changes in transcript and metabolite profiles. To date, the acclimation mechanisms that regulate light acclimation have been considered only in terms of immediate effects of light on the photosynthetic apparatus leading to chloroplast‐to‐nucleus signalling.

Photosynthesis rates were higher in tobacco leaves with low AO activity and a more reduced apoplastic redox state under HL growth conditions than in the leaves with high AO activity and a more oxidized apoplastic environment. Moreover, leaf AO activity and apoplastic redox state exerted a strong influence over a wide range of processes involved in the acclimation of leaves with a HL history to LL. These included lipid remodelling, amino acid metabolism and the levels of transcripts associated with protein turnover. Intracellular ROS production is considered to be a key component of the perception of changes in irradiance in leaves (Scheibe *et al*. 2005, Suzuki *et al*. 2012). While previous observations have shown that auxin‐induced ROS production in the apoplast exerts an influence over the induction phase of photosynthesis (Guo *et al*. 2016), the data presented here suggest that such changes in the redox state of the apoplast/cell wall compartment may also exert a strong influence over the light response and acclimation process.

The effects of light on the leaf transcriptome of Arabidopsis thaliana have been extensively characterized, with many genes encoding proteins involved in photosynthesis, particularly PSI and PSII components, being significantly more highly expressed in the light than in the dark or low illumination (Ma *et al*. 2001, Rossel *et al*. 2002). Light alters the expression of nuclear genes to a much greater extent than genes encoded by the plastid or mitochondrial genomes (Liang *et al*. 2016). The leaf transcript and metabolite profile data presented here broadly agree with the literature with regard to light‐mediated effects on gene expression and leaf metabolite profiles. However, the data show that AO activity and hence the redox state of the apoplast/cell wall compartment exert an influence over the acclimation of specific subsets of leaf transcripts and metabolite pools.

The evidence presented here indicates that AO activity also exerts an influence on the light acclimation process. Firstly, the levels of *β*‐Ala, Asp and Thr were much higher in PAO plants following the transition from HL to LL compared to those in plants that had been grown only under LL (Supporting Information Fig. S5). Chloroplasts are able to synthesize 17 protein amino acids from either Ala or Asp. The influence of a highly oxidized ascorbate pool in the apoplast on leaf Asp levels is important because the Asp‐derived amino acid pathway leads to the production of lysine (Lys), methionine (Met), Thr and Ile in the chloroplasts. Like Asp, Thr had much higher levels in the PAO plants than in the other lines, suggesting that the apoplastic redox state influences chloroplast amino acid synthesis. In contrast to Asp and Thr, *β*‐Ala is a non‐protein amino acid that is a precursor of pantothenate, which is itself a precursor of coenzyme A. It is produced by the reductive catabolism of pyrimidine nucleotides. The levels of *β*‐Ala, which can function as an ROS scavenger are often increased in response to environmental stresses such as HL and heat stress.

While transgenic tomato plants with low AO activity had higher leaf hexose and sugar contents (Garchery *et al*. 2013), these results were not consistently replicated in the present study. We observed a significant effect of plant genotype (TAO, WT and PAO) on the content of fructose following a recovery period under LL and on sucrose and glucose 6‐phosphate; however, both low‐AO and high‐AO lines exhibited higher content than did WT (Supporting Information Fig. S4). This effect was much lower than the impact of light, which tended to increase the content of sugars (Supporting Information Fig. S3). Moreover, AO activity had a significant effect on leaf fatty acid contents. Lipid remodelling, which is a key component of leaf acclimation to changing light levels, was also strongly influenced by AO activity, as evidenced by effects of AO on the abundance of several fatty acids (Supporting Information Fig. S4).

Ascorbate biosynthesis and accumulation are regulated by the quantity and quality of incident light perceived by the leaves (Yabuta *et al*. 2007, Bartoli *et al*. 2006, 2009). The data presented here show that the level of ascorbate in the leaves of all tobacco lines grown under LL was only about a third of that of plants grown under HL. Moreover, the leaves of plants transferred to LL retained a memory of the HL environment in terms of the level of leaf ascorbate accumulation over the whole of the first photoperiod; that is, ascorbate levels under LL conditions were consistent with the previous HL history of the leaves rather than the prevailing irradiance. Hence, leaf ascorbate was slow to acclimate after the transition to LL. The high levels of ascorbate cannot be explained in terms of altered expression of genes encoding enzymes of ascorbate synthesis. In fact, the transcriptome data presented here show that transcripts encoding guanosine diphosphate (GDP)‐d‐mannose 3,5‐epimerase (TA14362) and GDP‐l‐galactose phosphorylase (TA12116 and TA12117) were significantly lower following the transition from HL to LL than those in plants exposed to constant LL. The expression of ascorbate synthesis genes in *Arabidopsis* is under negative regulation by ascorbic acid mannose pathway regulator 1 (AMR1), the HL‐dependent repression of *AMR1* expression connecting the extent of ascorbate accumulation to light availability (Zhang *et al*. 2009). While the array data presented here do not reveal information concerning *AMR1* expression in tobacco, the findings show marked repression of genes encoding ascorbate synthesis enzymes following the transition to LL, consistent with observations in *Arabidopsis*. Hence, post‐transcriptional mechanisms are likely to link ascorbate synthesis and accumulation to the previous HL history of the leaves. The delay in the acclimation of the ascorbate pool to LL over a relatively short timescale may be of physiological advantage, as it would help protect photosynthesis against the negative impacts of sun flecks within the canopy. Interestingly, the extent of leaf ascorbate accumulation and maximal extractable AO activities increased throughout the photoperiod in a similar manner, suggesting a relationship between these parameters that has not been previously described. Both light intensity and time under light had a significant effect on maximal extractable AO activities of PAO and TAO leaves. An effect of light on AO activity has been reported previously (De Tullio *et al*. 2007). While a strong dark/light pattern of AO expression was observed in WT leaves (Pignocchi *et al*. 2003), the diurnal changes in AO activity that were observed in the PAO leaves, where AO expression is driven by the 35S promoter, strongly suggest that the activity of this enzyme is regulated at a post‐transcriptional level.

In contrast to the cytoplasm, the apoplastic/cell wall compartment has only relatively low levels of ascorbate (Pignocchi and Foyer 2003, Foyer and Noctor 2005, Zechmann, 2011). While the extent of leaf ascorbate accumulation and leaf ascorbate/DHA ratios were largely unaffected by changes in leaf AO activity, the ascorbate/DHA ratios of the apoplast and the levels of threonate, which is produced predominantly during ascorbate degradation, were strongly influenced by AO activity (Pignocchi and Foyer 2003, Sanmartin *et al*. 2003). The data presented here show that high AO activities also determine the extent of apoplastic ascorbate accumulation in a photoperiod‐dependent manner. While leaf maximal extractable AO activities firstly increased during the photoperiod in the PAO leaves and then decreased at the end of the photoperiod, the levels of ascorbate plus DHA in the apoplastic/cell wall compartment gradually increased throughout the photoperiod. The levels of ascorbate plus DHA also increased in the apoplastic/cell wall compartment of the leaves of other genotypes over the photoperiod, but these increases were 10‐fold higher in the PAO leaves.

In addition to changes in the availability of ascorbate and AO in the apoplastic/cell wall compartment, there are many enzymes that can induce apoplastic ROS production and hence lead to oxidation of the apoplast. These include the NADPH oxidases that catalyse hypersensitive response‐associated oxidative burst and cell‐wall‐localized peroxidases and oxidases such as oxalate oxidases that catalyse the conversion of oxalate to hydrogen peroxide and carbon dioxide (Zhu *et al*. 2007, Spollen *et al*. 2008, Davidson *et al*. 2009, Voothuluru and Sharp 2013). ROS fulfil essential roles in the apoplastic/cell wall compartment such as the regulation of growth, particularly with regard to cell wall loosening or tightening (Fry 1998, Cordoba‐Pedregosa *et al*. 2003, Foreman *et al*. 2003, Tyburski *et al*. 2010). Oxidative cleavage of cell wall polysaccharides is required for wall loosening and cell expansion (Schopfer 2001, Fry 2004, Muller *et al*. 2009), with the inhibition of hydroxyl radical production leading to an inhibition of growth in roots (Liszkay *et al*. 2004) and leaves (Rodriguez *et al*. 2002). Sucrose transport is also sensitive to the redox environment of the apoplastic/cell wall compartment. The SUT family of sucrose transporters interact with StPDI1, a protein disulphide isomerase (Eggert *et al.* 2016). *StPDI1*‐silenced plants have low ascorbate levels and show increased H_2_O_2_ accumulation under stress conditions (Eggert *et al*. 2016). However, it is worth noting that the action of AO may extend far beyond simple effects due to changes in AO activity. For example, expression of the rice AO protein, OsORAP1, which is localized in the apoplast but has no AO activity, enhanced cell death in leaves exposed to ozone or pathogens (Ueda *et al*. 2015). OsORAP1, whose expression was highest in photosynthetic tissues with the highest stomatal conductance, influences jasmonic acid pathway signalling to mitigate ozone symptoms by unknown mechanisms (Ueda *et al*. 2015).

**Figure 8:**
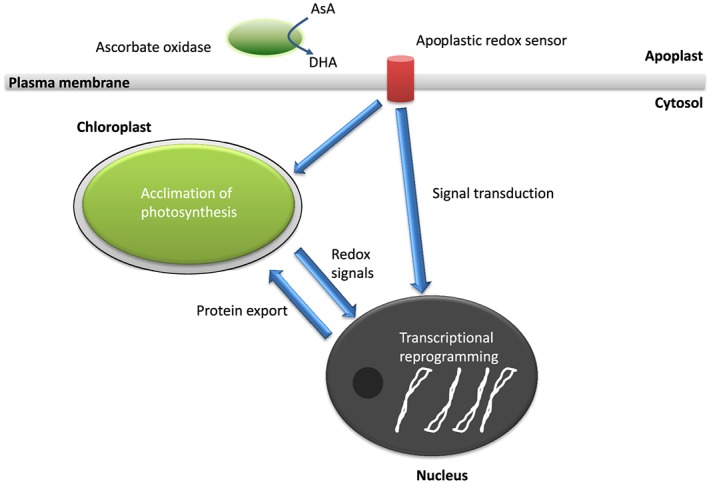
A model showing the role of apoplastic redox signalling in photosynthetic acclimation. Under a changing light environment, redox signals generated in the chloroplast are sensed in the nucleus, leading to transcriptional reprogramming and the import of chloroplast proteins to allow photosynthetic acclimation. Similarly, transcriptional reprogramming occurs within the plastid genome to aid with acclimation events. In the present manuscript, we demonstrate that apoplastic redox status also influences the process of acclimation to high light. Apoplastic redox state is determined in part by the ascorbate (AsA)/dehydroascorbate (DHA) ratio, which is regulated by AsA oxidase activity. We did not observe any changes in symplastic AsA/DHA ratios when AsA oxidase activity was modified. We conclude therefore that the apoplastic redox state exerts a strong direct influence on transcriptional reprogramming. Apoplastic redox signals are presumably sensed, and this information is communicated to the nucleus and chloroplasts to induce appropriate responses.

In conclusion, the data presented here demonstrate that the redox state of the apoplast plays a role in the acclimation of photosynthesis to changing irradiance, as illustrated in Fig. 8. The AO activity of the apoplast exerted an influence over the HL‐induced re‐organization of the leaf metabolome. Low AO activity resulted in a decrease in the susceptibility of photosynthesis to HL‐induced inhibition. The data presented here also show that the acclimation of ascorbate synthesis to LL is a relatively slow process in leaves. HL‐grown plants retained a high capacity for ascorbate synthesis, leading to greater leaf ascorbate levels during the first photoperiod following the transition to LL than those in plants grown only under LL. Moreover, data are presented showing that maximal extractable AO activity varied markedly during the photoperiod, particularly in the PAO lines, suggesting strong regulation of the activity of this enzyme at the post‐transcriptional level. These findings offer a significant new contribution to our current understanding of the role of the redox state of the apoplast in the regulation of tolerance to HL stress. While tobacco may not be considered to be a crop plant, the new knowledge gained through this research can readily be translated into crops or used in marker‐assisted selection for more climate‐resilient varieties.

### Author contributions

C.H.F. conceived the study. C.H.F., R.D.H. and P.E.H. designed experiments. B.R., B.K. and K.Z. conducted the experiments, except for the arrays, which were performed by P.E.H. and J.M. S.R.V. conducted statistical analysis. C.H.F. and R.D.H. wrote the manuscript with input from P.E.H.

## Supporting information


**Table S1**. Relative transcript abundance at the end of the HL period day 7. Transcripts specifically changed in abundance by growth irradiance were identified following microarray analysis. Transcripts exhibiting a minimum twofold change were then further classified using the MapMan tool. Transcripts are ordered according to their MapMan bin code and a description of the MapMan bin (bin name is also provided). ID refers to the probe identification number of the Agilent tobacco gene expression microarray (design ID 02113), and a brief description of the corresponding transcript is provided. Transcript abundance is represented as the fold change HL relative to LL (log_2_) for each genotype, and cells are coloured blue–red (high–low) for ease of reference.
**Table S2**. Relative transcript abundance at the end of the first LL period day 8. Transcripts specifically changed in abundance by growth irradiance history, genotype or an interaction of the two were identified following microarray analysis. Transcripts were then further classified using the MapMan tool, selecting only those that exhibited a twofold change dependent on irradiance history. Transcripts are ordered according to their MapMan bin code, and a description of the MapMan bin (bin name) is also provided. ID refers to the probe identification number of the Agilent tobacco gene expression microarray (design ID 02113), and a brief description of the corresponding transcript is provided. Transcript abundance is represented as the fold change HL relative to LL (log_2_) for each genotype, and cells are coloured blue–red (high–low) for ease of reference.
**Table S3**. Statistical significance of the factors light and genotype on tobacco leaf metabolite immediately after the end of 7 d of HL treatment (stress) or following return to LL for 12 h.
**Figure S1**. PageMan representation of gene expression data from plants harvested at the end of the final HL photoperiod. Data were expressed as log_2_ fold changes of plants exposed to HL relative to those grown under LL. Blue and red cells represent statistically significant increases or decreases in transcript abundance associated with specific functional categories according to the scale indicated. Headings to the left of the image represent major MapMan bins, while headings to the right indicate sub‐bins.
**Figure S2**. PageMan representation of gene expression data from plants harvested following an HL treatment and then a period of recovery under LL. Data were expressed as log_2_ fold changes in plants that experienced a HL episode relative to those grown solely under LL. Blue and red cells represent statistically significant increases or decreases in transcript abundance associated with specific functional categories according to the scale indicated. Headings to the left of the image represent major MapMan bins, while headings to the right indicate sub‐bins.
**Figure S3**. Influence of light regime on metabolites in tobacco leaves. Relative metabolite levels in leaves of TAO, WT and PAO tobacco plants following harvest at the end of the final HL photoperiod (black bars) or following 12 h recovery under LL (white bars) were estimated by GC/MS. Metabolites that exhibited a significant (*P* < 0.05) change in response to the light regime at either of the time points analysed as estimated by two‐way anova (light, genotype) are presented. Data are represented as mean ± SED, *n* = 3. An asterisk above the first bar indicates that specific differences at the end of the HL regime or following 12 h recovery, respectively.
**Figure S4**. Influence of genotype on metabolites in tobacco leaves. Relative metabolite levels in leaves of TAO, WT and PAO tobacco plants following harvest at the end of the final HL photoperiod (black bars) or following 12 h recovery under LL (white bars) were estimated by GC/MS. Metabolites that exhibited a significant (*P* < 0.05) difference dependent on genotype at either of the time points analysed as estimated by two‐way anova (light, genotype) are presented. Data are represented as mean ± SED, *n* = 3. An asterisk above the first bar indicates specific differences at the end of the HL regime or following 12 h recovery, respectively.
**Figure S5**. Genotype‐dependent influence of light on metabolites in tobacco leaves. Relative metabolite levels in leaves of TAO, WT and PAO tobacco plants following harvest at the end of the final HL photoperiod (black bars) or following 12 h recovery under LL (white bars) were estimated by GC/MS. Metabolites that exhibited a significant (*P* < 0.05) light‐dependent genotypic difference at either of the time points analysed as estimated by two‐way anova (light, genotype) are presented. Data are represented as mean ± SED, *n* = 3. An asterisk above the first bar indicates specific differences at the end of the HL regime or following 12 h recovery, respectively.

## References

[pce12960-bib-0001] Athanasiou K. , Dyson B.C. , Webster R.E. & Johnson G.N. (2010) Dynamic acclimation of photosynthesis increases plant fitness in changing environments. Plant Physiology 152, 366–373. 19939944 10.1104/pp.109.149351 PMC2799370

[pce12960-bib-0002] Bartoli C.G. , Gómez F. , Fernández L. , Yu J. , McIntosh L. & Foyer C.H. (2006) Inter‐relationships between light and respiration in the control of ascorbic acid synthesis and accumulation in Arabidopsis thaliana leaves. Journal of Experimental Botany 57, 1621–1631. 16714304 10.1093/jxb/erl005

[pce12960-bib-0003] Bartoli C. , Tambussi E.A. , Diego F. & Foyer C.H. (2009) Control of ascorbic acid synthesis and accumulation and glutathione by the incident light red/far red ratio in Phaseolus vulgaris leaves. FEBS Letters 583, 118–122. 19059408 10.1016/j.febslet.2008.11.034

[pce12960-bib-0004] Chenier V. , Comte G. , Favies K.M. , Lattanzio V. & Martens S. (2013) Plant phenolics: recent advances on their biosynthesis, genetics, and ecophysiology. Plant Physiology and Biochemistry 72, 1–20. 23774057 10.1016/j.plaphy.2013.05.009

[pce12960-bib-0005] Cohu C.M. , Muller O. , Adams W.W. & Demmig‐Adams B. (2014) Leaf anatomical and photosynthetic acclimation to cool temperature and high light in two winter versus two summer annuals. Physiologia Plantarum 152, 164–173. 24450735 10.1111/ppl.12154

[pce12960-bib-0006] Cordoba‐Pedregosa M. , Cordoba F. , Villalba J.M. & Gonzalez‐Reyes J.A. (2003) Zonal changes in ascorbate and hydrogen peroxide contents, peroxidase, and ascorbate‐related enzyme activities in onion roots. Plant Physiology 131, 697–706. 12586893 10.1104/pp.012682 PMC166845

[pce12960-bib-0007] Davidson R.M. , Reeves P.A. , Manosalva P.M. & Leach J.E. (2009) Germins: a diverse protein family important for crop improvement. Plant Science 177, 499–510.

[pce12960-bib-0008] Demmig‐Adams B. , Stewart J.J. & Adams W.W. (2014) Multiple feedbacks between chloroplast and whole plant in the context of plant adaptation and acclimation to the environment. Philosophical Transactions of the Royal Society B 269, 20130244. 10.1098/rstb.2013.0244 PMC3949402 24591724

[pce12960-bib-0009] De Tullio M.C. , Guether M. & Balestrini R. (2013) Ascorbate oxidase is the potential conductor of a symphony of signaling pathways. Plant Signaling and Behavior 8, e23213. 10.4161/psb.23213 PMC3676494 23299329

[pce12960-bib-0010] De Tullio M.C. , Ciraci S. , Liso R. & Arrigoni O. (2007) Ascorbic acid oxidase is dynamically regulated by light and oxygen. A tool for oxygen management in plants? Journal of Plant Physiology 164, 39–46. 16343690 10.1016/j.jplph.2005.09.016

[pce12960-bib-0011] Eggert E. , Obata T. , Gerstenberger A. , Gier K. , Brandt T. , Fernie A.R. , Schulze W. & Kühn K. (2016) A sucrose transporter‐interacting protein disulphide isomerase affects redox homeostasis and links sucrose partitioning with abiotic stress tolerance. Plant Cell and Environment 39, 1366–1380. 10.1111/pce.12694 26670204

[pce12960-bib-0012] Foito A. , Byrne S.L. , Hackett C.A. , Hancock R.D. , Stewart D. & Barth S. (2013) Short‐term response in leaf metabolism of perennial ryegrass (Lolium perenne) to alterations in nitrogen supply. Metabolomics 9, 145–156.

[pce12960-bib-0013] Foreman J. , Demidchik V. , Bothwell J.H.F. , Mylona P. , Miedema H. , Torres M.A. , … Dolan L. (2003) Reactive oxygen species produced by NADPH oxidase regulate plant cell growth. Nature 422, 442–446. 12660786 10.1038/nature01485

[pce12960-bib-0014] Foyer C.H. & Noctor G. (2005) Redox homeostasis and antioxidant signalling: a metabolic interface between stress perception and physiological responses. Plant Cell 17, 1866–1875. 15987996 10.1105/tpc.105.033589 PMC1167537

[pce12960-bib-0015] Foyer C.H. , Rasool B. , Davey J. & Hancock R.D. (2016) Cross tolerance to biotic and abiotic stresses in plants: a focus on resistance to aphid infestation. Journal of Experimental Botany 67, 2025–2037. 26936830 10.1093/jxb/erw079

[pce12960-bib-0016] Fry S.C. (1998) Oxidative scission of plant cell wall polysaccharides by ascorbate‐induced hydroxyl radicals. Biochemical Journal 332, 507–515. 9601081 10.1042/bj3320507 PMC1219507

[pce12960-bib-0017] Fry S.C. (2004) Primary cell wall metabolism: tracking the careers of wall polymers in living plant cells. New Phytologist 161, 641–675. 10.1111/j.1469-8137.2004.00980.x 33873719

[pce12960-bib-0018] Garchery C. , Gest N. , Do P.T. , Alhagdow M. , Baldet P. , Menard G. , … Stevens R. (2013) A diminution in ascorbate oxidase activity affects carbon allocation and improves yield in tomato under water deficit. Plant Cell and Environment 36, 159–175. 10.1111/j.1365-3040.2012.02564.x 22725103

[pce12960-bib-0019] Gilroy S. , Suzuki N. , Miller G. , Choi W.‐G. , Toyota M. , Devireddy A.R. & Mittler R. (2014) A tidal wave of signals: calcium and ROS at the forefront of rapid systemic signaling. Trends in Plant Science 19, 623–630. 25088679 10.1016/j.tplants.2014.06.013

[pce12960-bib-0020] Grace S.C. & Logan B.A. (1996) Acclimation of foliar antioxidant systems to growth irradiance in 3 broad‐leaved evergreen species. Plant Physiology 112, 1631–1640. 12226469 10.1104/pp.112.4.1631 PMC158097

[pce12960-bib-0021] Green B.R. , Anderson J.M. & Parson W.W. (2003) Photosynthetic membranes and their light‐harvesting antennas. Advances in Photosynthesis and Respiration 13, 1–28.

[pce12960-bib-0022] Green M.A. & Fry S.C. (2005) Vitamin C degradation in plant cells via enzymatic hydrolysis of 4‐*O*‐oxalyl‐l‐threonate. Nature 433, 83–87. 15608627 10.1038/nature03172

[pce12960-bib-0023] Guo Z. , Li H. , Xiang X. , Ahammed G.J. , Wang M. , Onac E. , … Zhou Y. (2016) Systemic induction of photosynthesis via illumination of the shoot apex is mediated sequentially by phytochrome B, auxin and hydrogen peroxide in tomato. Plant Physiology 172, 1259–1272. 27550998 10.1104/pp.16.01202 PMC5047115

[pce12960-bib-0024] Leakey A.D.B. , Scholes J.D. & Press M.C. (2005) Physiological and ecological significance of sunflecks for dipterocarp seedlings. Journal of Experimental Botany 56, 469–482. 15596478 10.1093/jxb/eri055

[pce12960-bib-0025] Liang C. , Cheng S. , Zhang Y. , Sun Y. , Fernie A.R. , Kang K. , … Lim B.L. (2016) Transcriptomic, proteomic and metabolic changes in Arabidopsis thaliana leaves after the onset of illumination. BMC Plant Biology 16, 43. 26865323 10.1186/s12870-016-0726-3 PMC4750186

[pce12960-bib-0026] Lichtenthaler H.K. (1986) Chlorophylls and carotenoids: pigments of photosynthetic biomembranes. Methods in Enzymology 148, 350–382.

[pce12960-bib-0027] Liszkay A. , van der Zalm E. & Schopfer P. (2004) Production of reactive oxygen intermediates (O_2_ ^·−^, H_2_O_2_, and ^·^OH) by maize roots and their role in wall loosening and elongation growth. Plant Physiology 136, 3114–3123. 15466236 10.1104/pp.104.044784 PMC523372

[pce12960-bib-0028] Ma L. , Li J. , Qu L. , Hager J. , Chen Z. , Zhao H. & Deng X.W. (2001) Light control of *Arabidopsis* development entails coordinated regulation of genome expression and cellular pathways. Plant Cell 13, 2589–2607. 11752374 10.1105/tpc.010229 PMC139475

[pce12960-bib-0029] Muller K. , Linkies A. , Vreeburg R.A.M. , Fry S.C. , Krieger‐Liszkay A. & Leubner‐Metzger G. (2009) *In vivo* cell wall loosening by hydroxyl radicals during cress seed germination and elongation growth. Plant Physiology 150, 1855–1865. 19493972 10.1104/pp.109.139204 PMC2719145

[pce12960-bib-0030] Muller O. , Cohu C.M. , Stewart J.J. , Protheroe J.A. , Demmig‐Adams B. & Adams W.W. (2014) Association between photosynthesis and contrasting features of minor veins in leaves of summer annuals loading phloem via symplastic versus apoplastic routes. Physiologia Plantarum 152, 174–183. 24450755 10.1111/ppl.12155

[pce12960-bib-0031] Noctor G. , Mhamdi A. & Foyer C.H. (2016) Oxidative stress and antioxidative systems: recipes for successful data collection and interpretation. Plant Cell and Environment 39, 1140–1160. 10.1111/pce.12726 26864619

[pce12960-bib-0032] Pignocchi C. , Fletcher J.M. , Wilkinson J.E. , Barnes J.D. & Foyer C.H. (2003) The function of ascorbate oxidase in tobacco. Plant Physiology 132, 1631–1641. 12857842 10.1104/pp.103.022798 PMC167100

[pce12960-bib-0033] Pignocchi C. & Foyer C.H. (2003) Apoplastic ascorbate metabolism and its role in the regulation of cell signalling. Current Opinion in Plant Biology 6, 379–389. 12873534 10.1016/s1369-5266(03)00069-4

[pce12960-bib-0034] Pignocchi C. , Kiddle G. , Hernández I. , Foster S.J. , Asensi A. , Taybi T. , Barnes J. & Foyer C.H. (2006) Ascorbate oxidase‐dependent changes in the redox state of the apoplast modulate gene transcript accumulation leading to modified hormone signaling and orchestration of defense processes in tobacco. Plant Physiology 141, 423–435. 16603663 10.1104/pp.106.078469 PMC1475448

[pce12960-bib-0035] Queval G. & Noctor G. (2007) A plate reader method for the measurement of NAD, NADP, glutathione and ascorbate in tissue extracts: application to redox profiling during *Arabidopsis* rosette development. Analytical Biochemistry 363, 58–69. 17288982 10.1016/j.ab.2007.01.005

[pce12960-bib-0036] Rodriguez A.A. , Grunberg K.A. & Taleisnik E.L. (2002) Reactive oxygen species in the elongation zone of maize leaves are necessary for leaf extension. Plant Physiology 129, 1627–1632. 12177475 10.1104/pp.001222 PMC166750

[pce12960-bib-0037] Rossel J.B. , Wilson I.W. & Pogson B.J. (2002) Global changes in gene expression in response to high light in *Arabidopsis* . Plant Physiology 130, 1109–1120. 12427978 10.1104/pp.005595 PMC166632

[pce12960-bib-0038] Sanmartin M. , Drogoudi P.A. , Lyons T. , Pateraki I. , Barnes J. & Kanellis A.K. (2003) Over‐expression of ascorbate oxidase in the apoplast of transgenic tobacco results in altered ascorbate and glutathione redox states and increased sensitivity to ozone. Planta 216, 918–928. 12687359 10.1007/s00425-002-0944-9

[pce12960-bib-0039] Scheibe R. , Backhausen J.E. , Emmerlich V. & Holtgrefe S. (2005) Strategies to maintain redox homeostasis during photosynthesis under changing conditions. Journal of Experimental Botany 56, 1481–1489. 15851411 10.1093/jxb/eri181

[pce12960-bib-0040] Schopfer P. (2001) Hydroxyl radical‐induced cell‐wall loosening *in vitro* and *in vivo*: implications for the control of elongation growth. Plant Journal 28, 679–688. 11851914 10.1046/j.1365-313x.2001.01187.x

[pce12960-bib-0041] Smirnoff N. & Pallanca J.E. (1996) Ascorbate metabolism in relation to oxidative stress. Biochemical Society Transactions 24, 472–478. 8736787 10.1042/bst0240472

[pce12960-bib-0042] Spollen W.G. , Tao W. , Valliyodan B. , Chen K. , Hejlek L.G. , Kim J.J. , … Nguyen H.T. (2008) Spatial distribution of transcript changes in the maize primary root elongation zone at low water potential. BMC Plant Biology 8, 32. 18387193 10.1186/1471-2229-8-32 PMC2364623

[pce12960-bib-0043] Suzuki N. , Koussevitzky S. , Mittler R. & Miller G. (2012) ROS and redox signalling in the response of plants to abiotic stress. Plant Cell and Environment 35, 259–270. 10.1111/j.1365-3040.2011.02336.x 21486305

[pce12960-bib-0045] Tyburski J. , Dunajska K. & Tretyn A. (2010) A role for redox factors in shaping root architecture under phosphorus deficiency. Plant Signaling and Behavior 5, 64–66. 20592813 10.4161/psb.5.1.10199 PMC2835962

[pce12960-bib-0046] Ueda Y. , Siddique S. & Frei M. (2015) A novel gene, OZONE‐RESPONSIVE APOPLASTIC PROTEIN1, enhances cell death in ozone stress in rice. Plant Physiology 169, 873–889. 26220952 10.1104/pp.15.00956 PMC4577431

[pce12960-bib-0047] Usadel B. , Nagel A. , Steinhauser D. , Gibon Y. , Bläsing O.E. , Redestig H. , Sreenivasulu N. & Stitt M. (2006) PageMan: an interactive ontology tool to generate, display, and annotate overview graphs for profiling experiments. BMC Bioinformatics 7, 535. 17176458 10.1186/1471-2105-7-535 PMC1766370

[pce12960-bib-0048] Voothuluru P. & Sharp R.E. (2013) Apoplastic hydrogen peroxide in the growth zone of the maize primary root under water stress. I. Increased levels are specific to the apical region of growth maintenance. Journal of Experimental Botany 64, 1223–1233. 23071257 10.1093/jxb/ers277

[pce12960-bib-0049] Walters R.G. (2005) Towards an understanding of photosynthetic acclimation. Journal of Experimental Botany 56, 435–447. 15642715 10.1093/jxb/eri060

[pce12960-bib-0050] Walters R.G. & Horton P. (1995a) Acclimation of Arabidopsis thaliana to the light environment – changes in photosynthetic function. Planta 197, 306–312. 8547817 10.1007/BF00202652

[pce12960-bib-0051] Walters R.G. & Horton P. (1995b) Acclimation of Arabidopsis thaliana to the light environment – regulation of chloroplast composition. Planta 197, 475–481. 8580761 10.1007/BF00196669

[pce12960-bib-0052] Yabuta Y. , Mieda T. , Rapolu M. , Nakamura A. , Motoki T. , Maruta T. , … Shigeoka S. (2007) Light regulation of ascorbate biosynthesis is dependent on the photosynthetic electron transport chain but independent of sugars in *Arabidopsis* . Journal of Experimental Botany 58, 2661–2671. 17586607 10.1093/jxb/erm124

[pce12960-bib-0053] Yamamoto A. , Bhuiyan M.N. , Waditee R. , Tanaka Y. , Esaka M. , Oba K. , Jagendorf A.T. & Takabe T. (2005) Suppressed expression of the apoplastic ascorbate oxidase gene increases salt tolerance in tobacco and *Arabidopsis* plants. Journal of Experimental Botany 56, 1785–1796. 15883131 10.1093/jxb/eri167

[pce12960-bib-0054] Yin Z.H. & Johnson G.N. (2000) Photosynthetic acclimation of higher plants to growth in fluctuating light environments. Photosynthesis Research 63, 97–107. 16252168 10.1023/A:1006303611365

[pce12960-bib-0055] Zechmann B. (2011) Subcellular distribution of ascorbate in plants. Plant Signaling and Behavior 6, 360–363. 21350341 10.4161/psb.6.3.14342 PMC3142415

[pce12960-bib-0056] Zhang W. , Lorence A. , Gruszewski H.A. , Chevone B.I. & Nessler C.L. (2009) *AMR1*, an *Arabidopsis* gene that coordinately and negatively regulates the mannose/l‐galactose ascorbic acid biosynthetic pathway. Plant Physiology 150, 942–950. 19395407 10.1104/pp.109.138453 PMC2689990

[pce12960-bib-0057] Zhu J.M. , Alvarez S. , Marsh E.L. , LeNoble M.E. , Cho I.J. , Sivaguru M. & Sharp R.E. (2007) Cell wall proteome in the maize primary root elongation zone. II. Region‐specific changes in water soluble and lightly ionically bound proteins under water deficit. Plant Physiology 145, 1533–1548. 17951457 10.1104/pp.107.107250 PMC2151692

