# Supplementary material for: The redox state of the apoplast influences the acclimation of photosynthesis and leaf metabolism to changing irradiance
Source: Plant Cell Environ. 2017 May 23;41(5):1083–97. doi: 10.1111/pce.12960 (PMC5947596; doi:10.1111/pce.12960)

Figure S3. Leaf content of metabolites significantly altered by light environment

Carbohydrates

Glucose peak 1

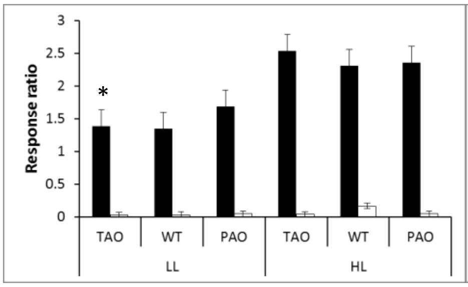

Glucose peak 2

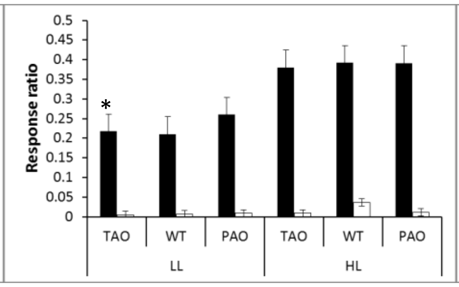

Unoximated fructose

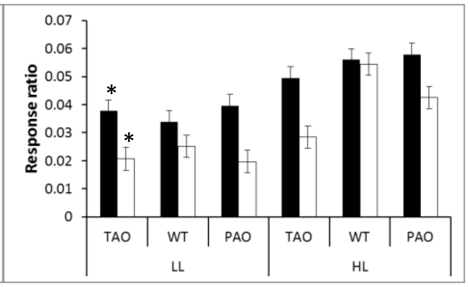

Sucrose

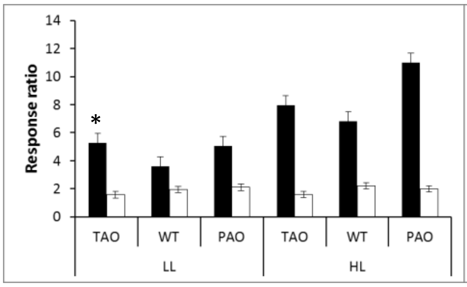

Glucose-6-P

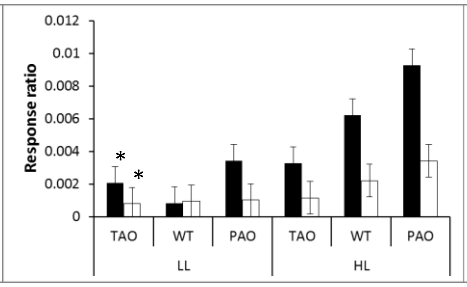

Fructose-6-P

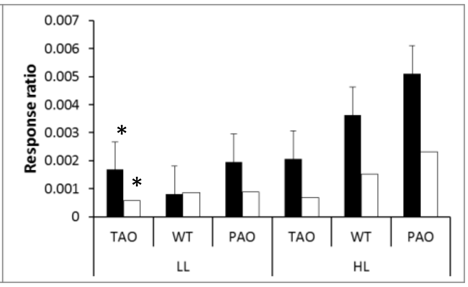

Dihydroxydihydrofuranone

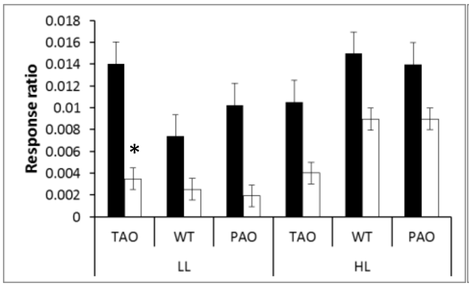

Unknown carbohydrate

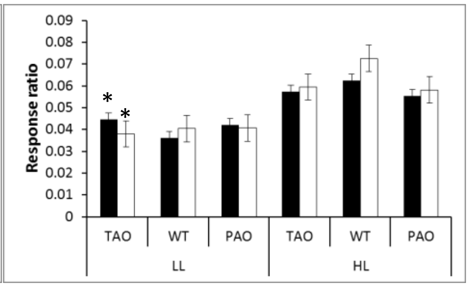

Organic acids

Succinate

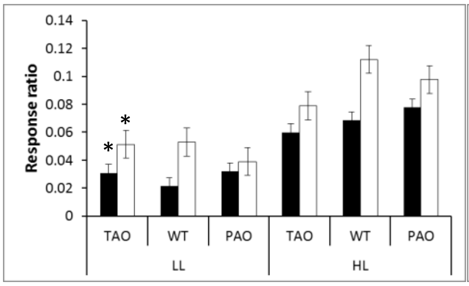

Fumarate

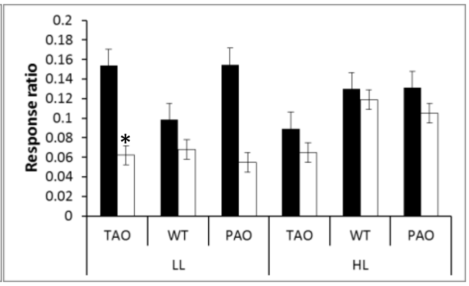

Malate

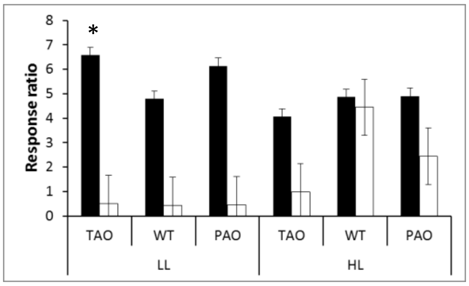

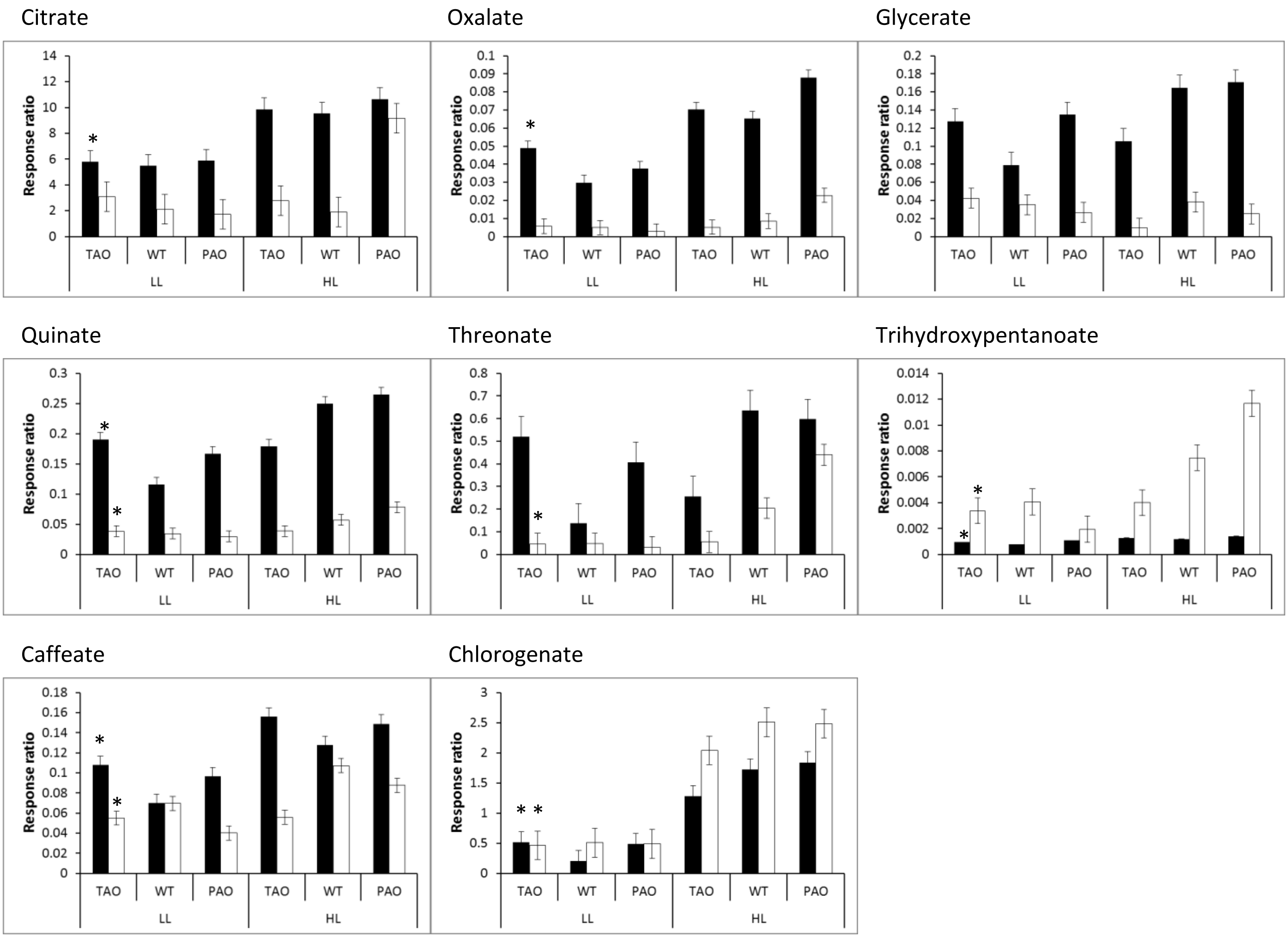

Amino acids and amines

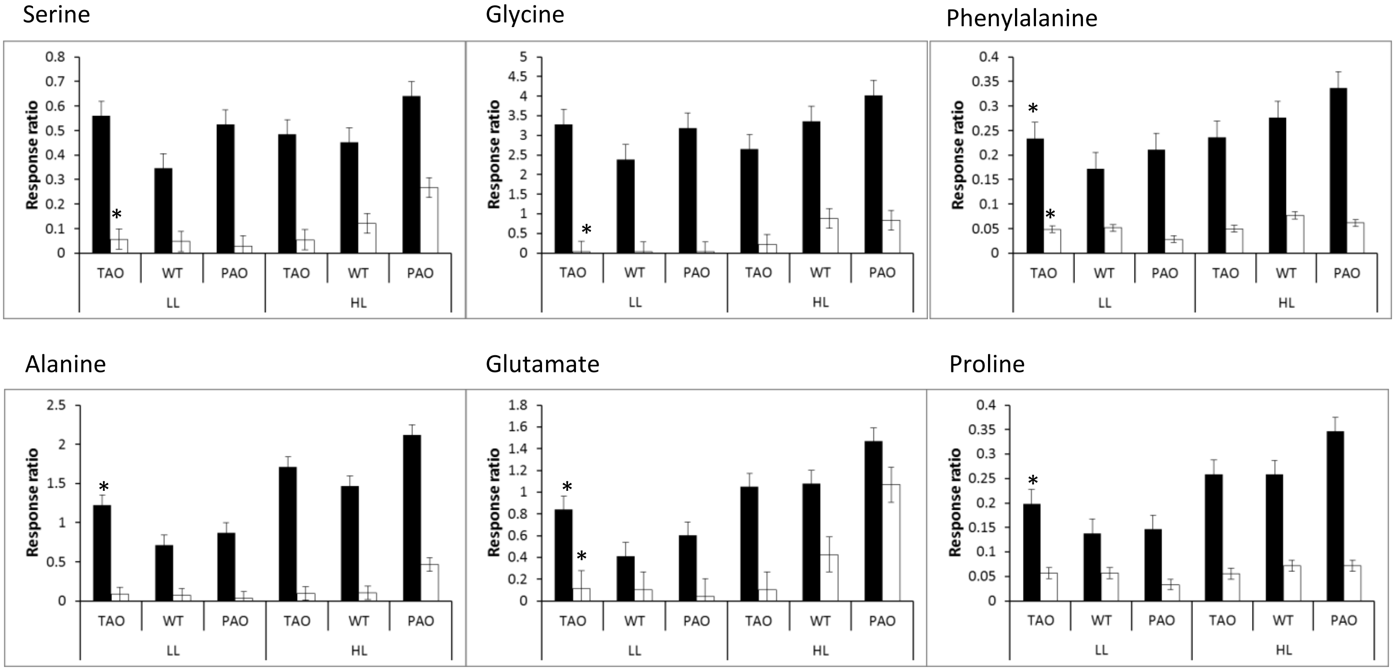

Hydroxy/Oxoproline

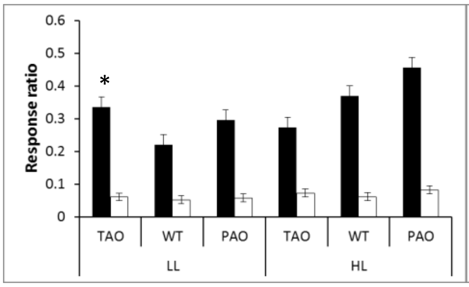

Leucine

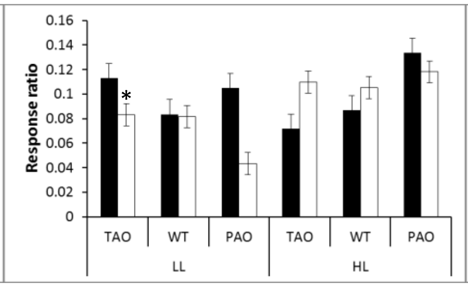

Valine

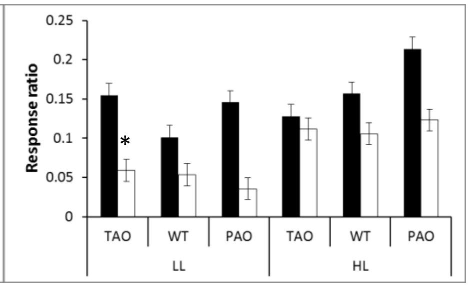

Aspartate

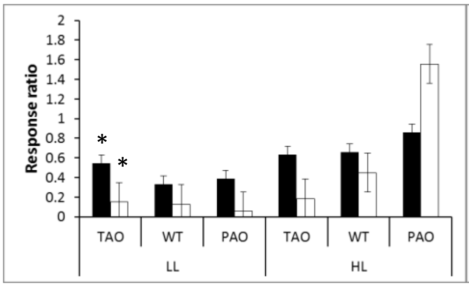

Threonine

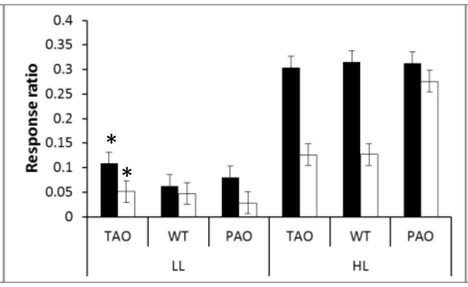

Isoleucine

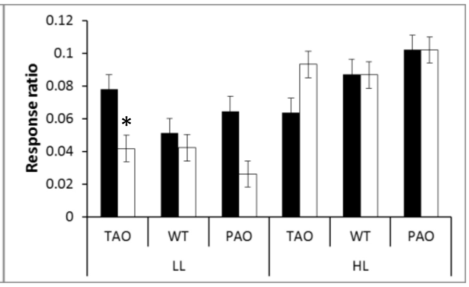

β-Alanine

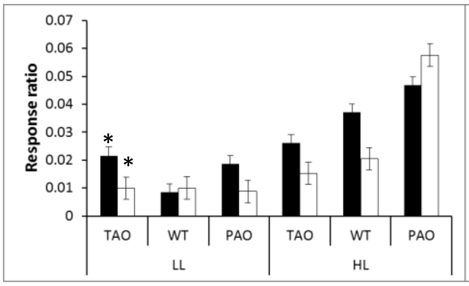

Putrescine

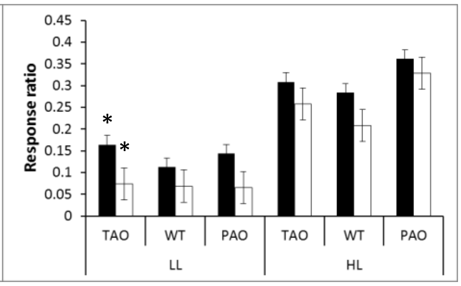

Fatty acids

Pentadecylate (C15:0)

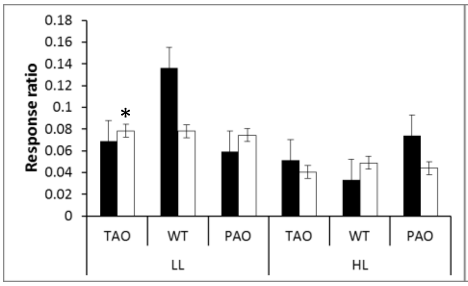

Methyl pentadecylate

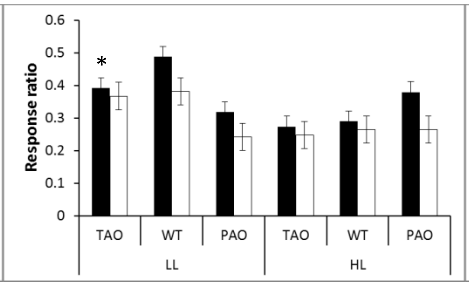

Methyl palmitate

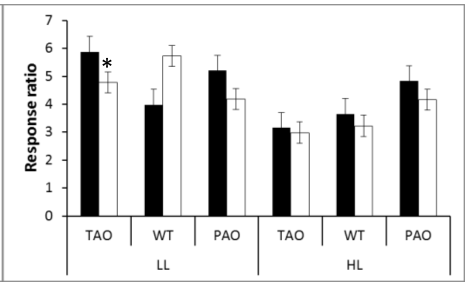

Palmitoleate (C16:1)

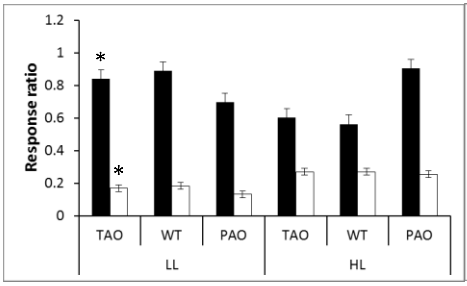

Margarate (C17:0)

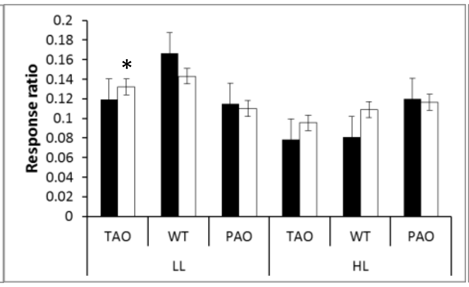

Oleate (C18:1)

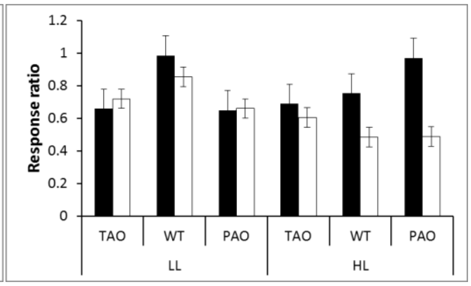

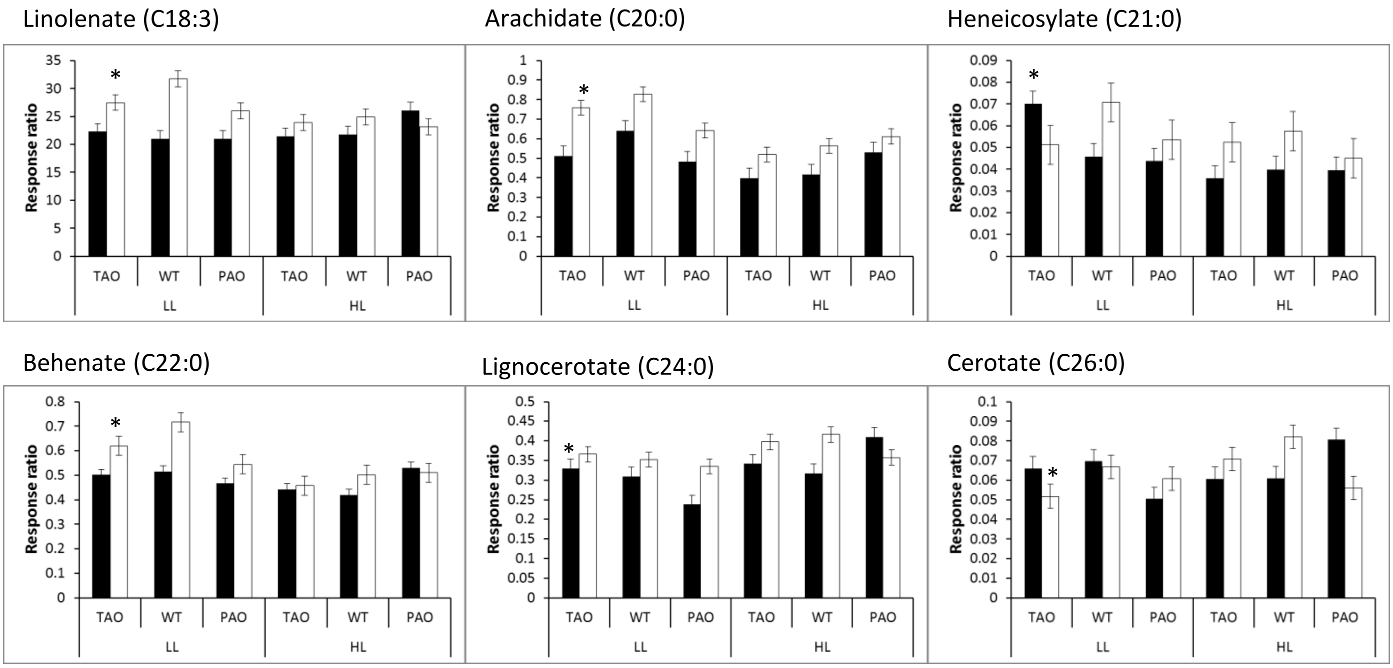

Fatty alcohols and phytosterols

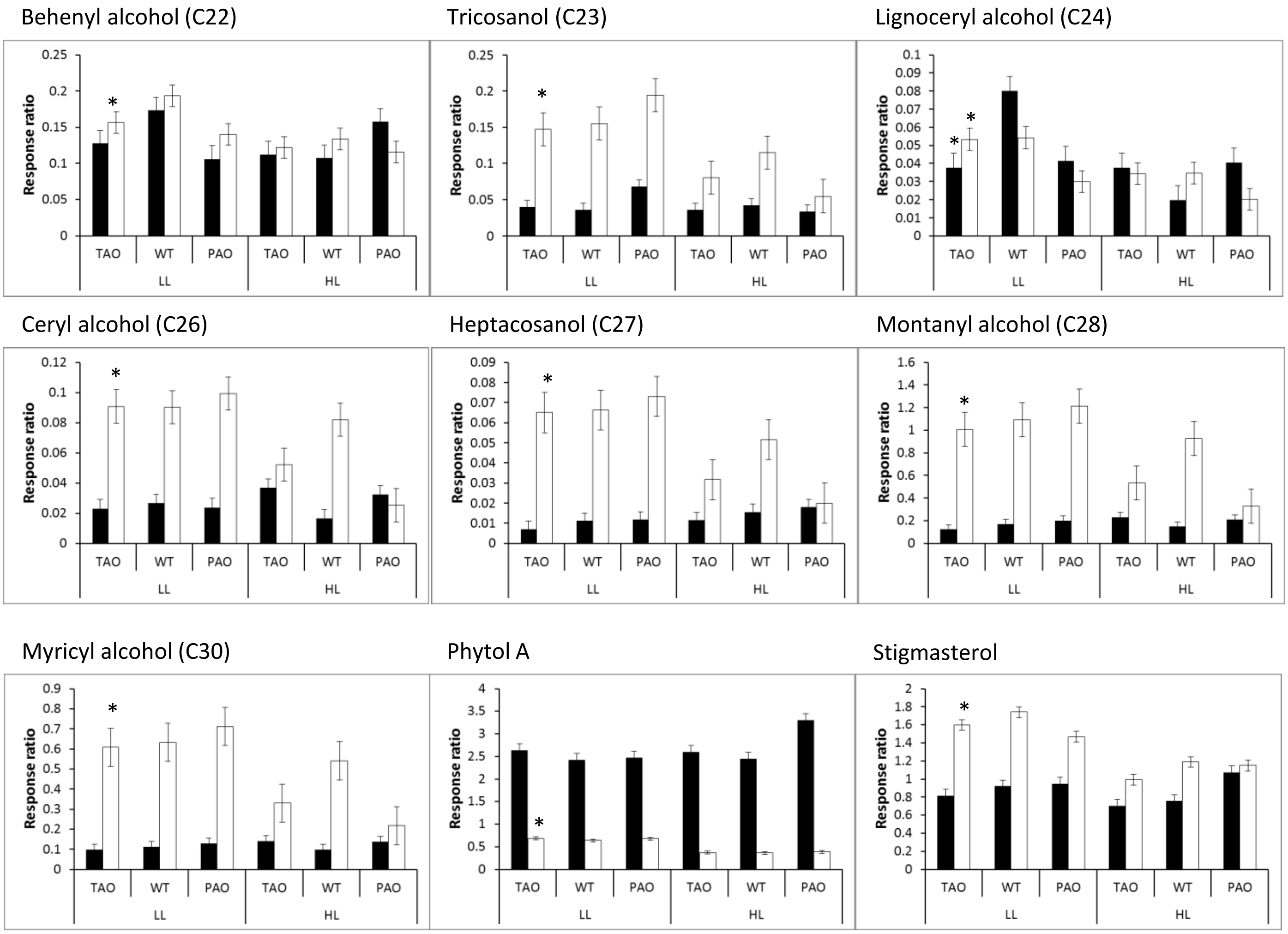

β-Sitosterol

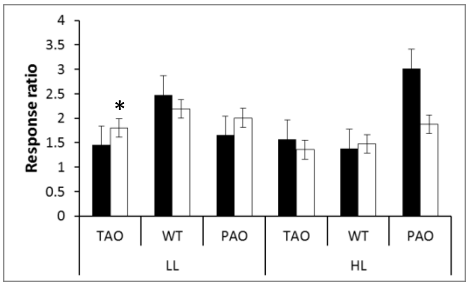

Miscellaneous and unknowns

Non-polar 8.12 min

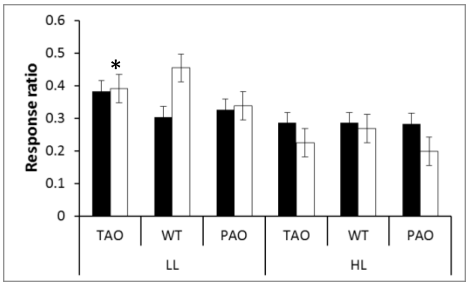

Supplement: Supplementary file 1 — Table S1. Relative transcript abundance at the end of the HL period day 7. Transcripts specifically changed in abundance by growth irradiance were identified following microarray analysis. Transcripts exhibiting a minimum twofold change were then further classified using the MapMan tool. Transcripts are ordered according to their MapMan bin code and a description of the MapMan bin (bin name is also provided). ID refers to the probe identification number of the Agilent tobacco gene expression microarray (design ID 02113), and a brief description of the corresponding transcript is provided. Transcript abundance is represented as the fold change HL relative to LL (log2) for each genotype, and cells are coloured blue–red (high–low) for ease of reference. Table S2. Relative transcript abundance at the end of the first LL period day 8. Transcripts specifically changed in abundance by growth irradiance history, genotype or an interaction of the two were identified following microarray analysis. Transcripts were then further classified using the MapMan tool, selecting only those that exhibited a twofold change dependent on irradiance history. Transcripts are ordered according to their MapMan bin code, and a description of the MapMan bin (bin name) is also provided. ID refers to the probe identification number of the Agilent tobacco gene expression microarray (design ID 02113), and a brief description of the corresponding transcript is provided. Transcript abundance is represented as the fold change HL relative to LL (log2) for each genotype, and cells are coloured blue–red (high–low) for ease of reference. Table S3. Statistical significance of the factors light and genotype on tobacco leaf metabolite immediately after the end of 7 d of HL treatment (stress) or following return to LL for 12 h. Figure S1. PageMan representation of gene expression data from plants harvested at the end of the final HL photoperiod. Data were expressed as log2 fold changes of plants exposed to HL [file PCE-41-1083-s001.zip › Figure S3.pdf]
