# Supplementary material for: The redox state of the apoplast influences the acclimation of photosynthesis and leaf metabolism to changing irradiance
Source: Plant Cell Environ. 2017 May 23;41(5):1083–97. doi: 10.1111/pce.12960 (PMC5947596; doi:10.1111/pce.12960)

Figure S5. Leaf content of metabolites significantly altered by light in a plant genotype dependent manner

Carbohydrates

Dihydroxydihydrofuranone

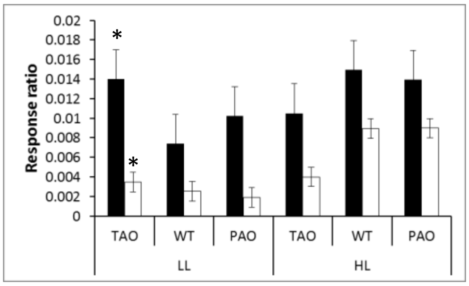

Organic acids

Malate

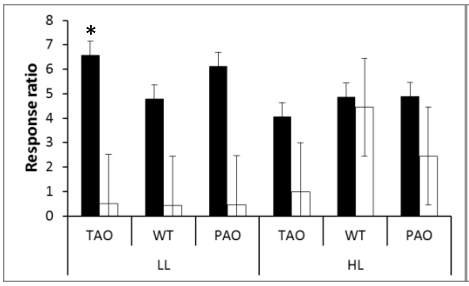

Citrate

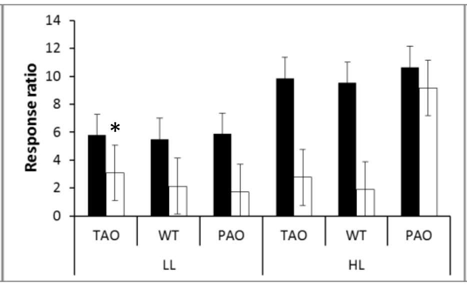

Glycerate

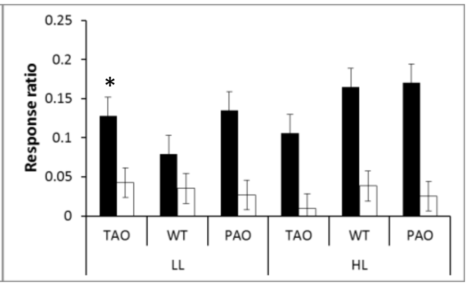

Quinate

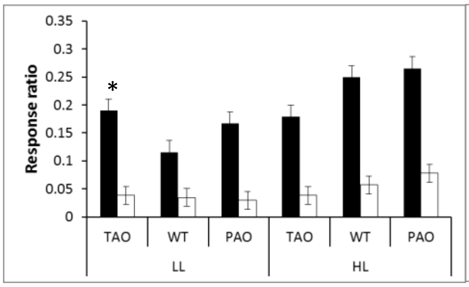

Threonate

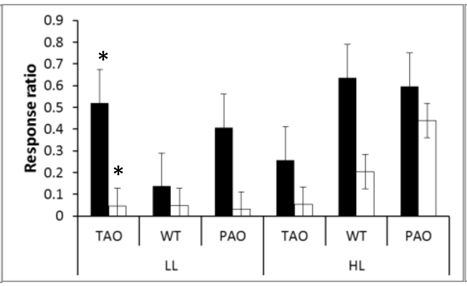

Trihydroxypentanoate

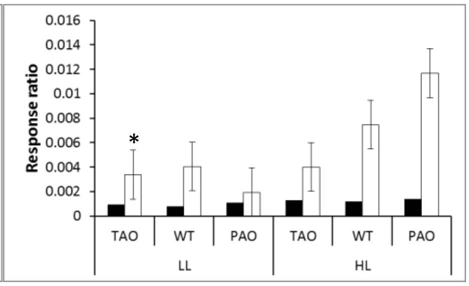

Caffeate

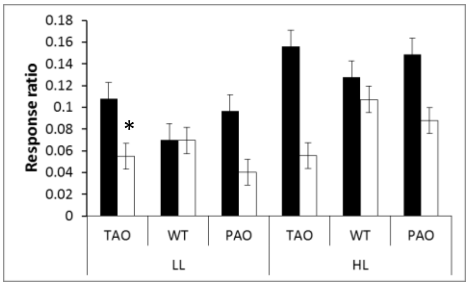

Amino acids and amines

Oxoproline

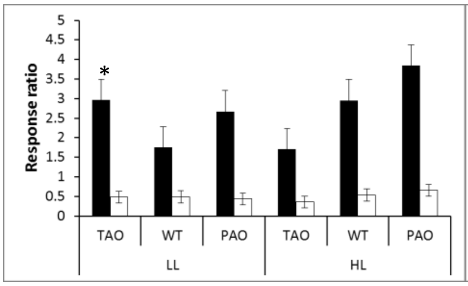

Hydroxy/Oxoproline

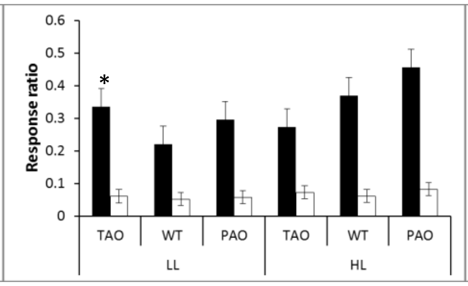

Aspartate

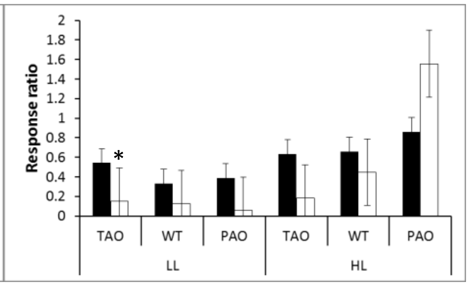

Threonine

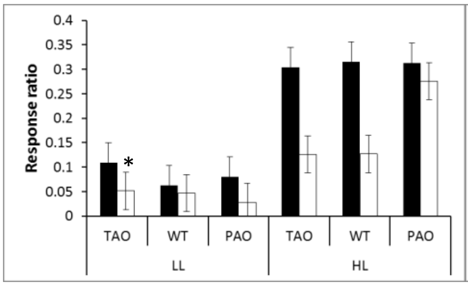

β-Alanine

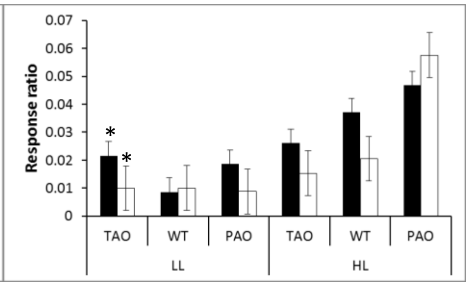

Fatty acids

Methyl pentadecylate

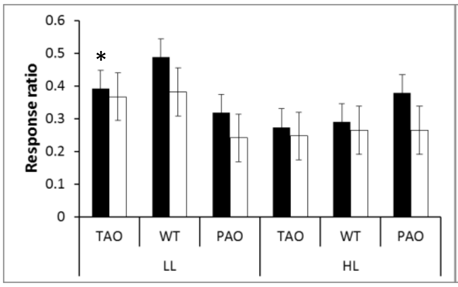

Palmitoleate (C16:1)

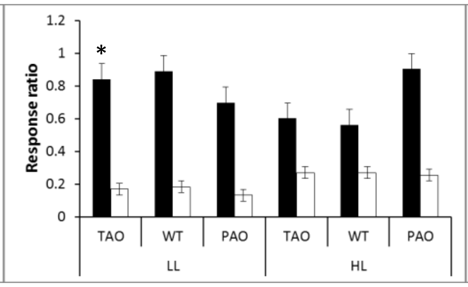

Sterate (C18:0)

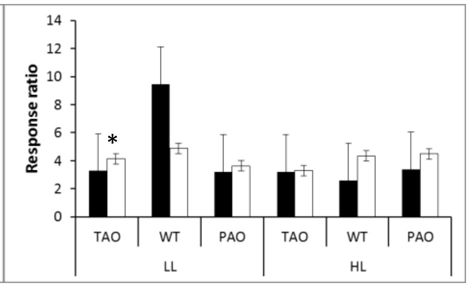

Linoleate (C18:2)

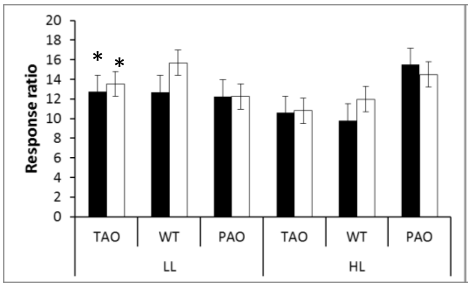

Arachidate (C20:0)

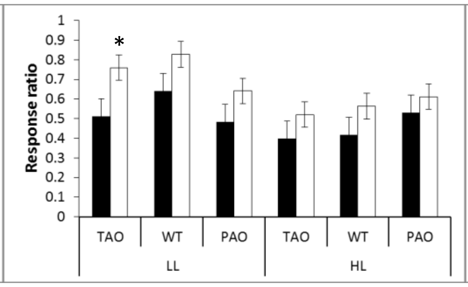

Behenate (C22:0)

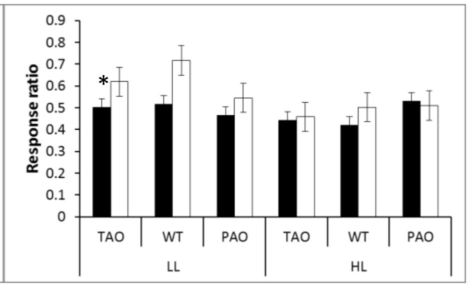

Tricosylate (C23:0)

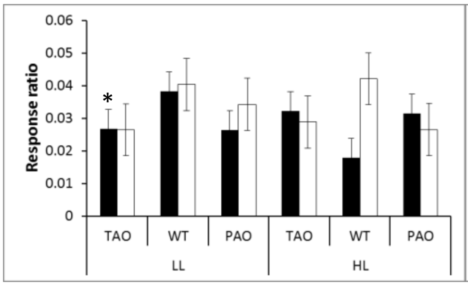

Lignocerate (C24:0)

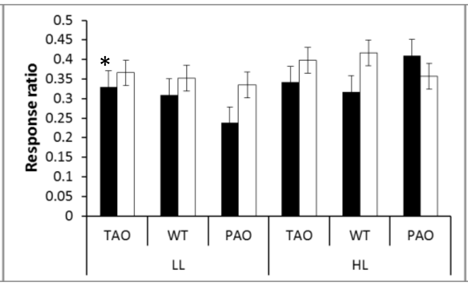

Hydroxylignocerate (C24:0)

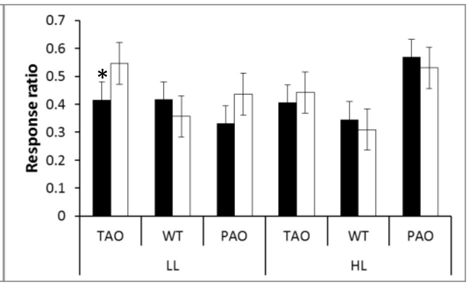

Cerotate (C26:0)

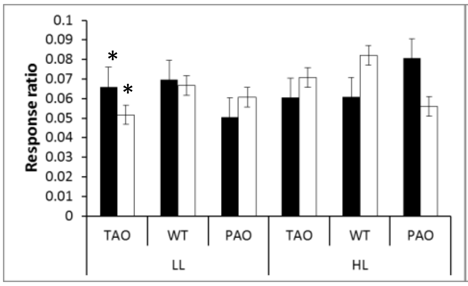

Montanate (C28:0)

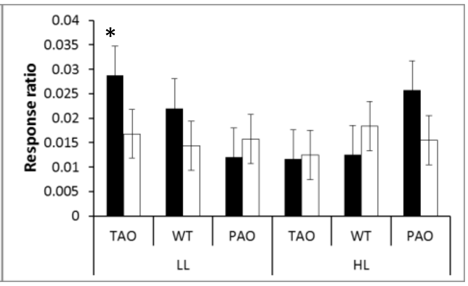

Fatty alcohols and phytosterols

Heneicosanol (C21)

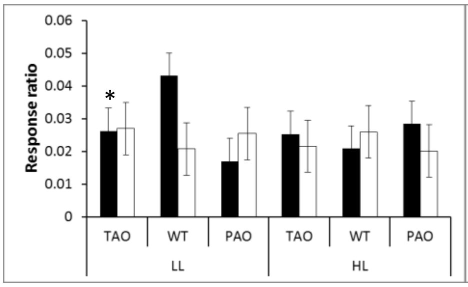

Lignoceryl alcohol (C24)

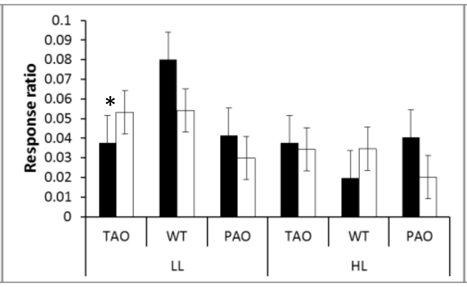

Phytol A

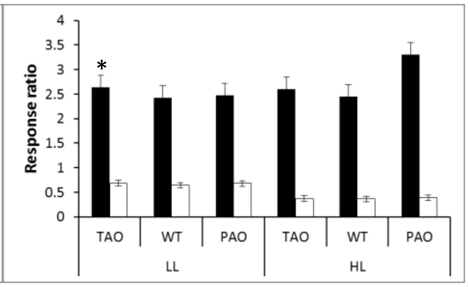

Miscellaneous and unknowns

Phosphate

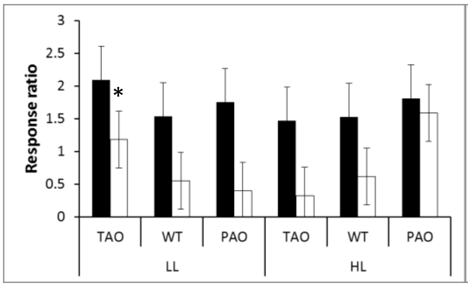

Polar 3.11 min

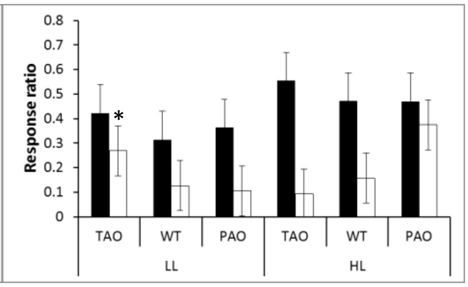

Supplement: Supplementary file 1 — Table S1. Relative transcript abundance at the end of the HL period day 7. Transcripts specifically changed in abundance by growth irradiance were identified following microarray analysis. Transcripts exhibiting a minimum twofold change were then further classified using the MapMan tool. Transcripts are ordered according to their MapMan bin code and a description of the MapMan bin (bin name is also provided). ID refers to the probe identification number of the Agilent tobacco gene expression microarray (design ID 02113), and a brief description of the corresponding transcript is provided. Transcript abundance is represented as the fold change HL relative to LL (log2) for each genotype, and cells are coloured blue–red (high–low) for ease of reference. Table S2. Relative transcript abundance at the end of the first LL period day 8. Transcripts specifically changed in abundance by growth irradiance history, genotype or an interaction of the two were identified following microarray analysis. Transcripts were then further classified using the MapMan tool, selecting only those that exhibited a twofold change dependent on irradiance history. Transcripts are ordered according to their MapMan bin code, and a description of the MapMan bin (bin name) is also provided. ID refers to the probe identification number of the Agilent tobacco gene expression microarray (design ID 02113), and a brief description of the corresponding transcript is provided. Transcript abundance is represented as the fold change HL relative to LL (log2) for each genotype, and cells are coloured blue–red (high–low) for ease of reference. Table S3. Statistical significance of the factors light and genotype on tobacco leaf metabolite immediately after the end of 7 d of HL treatment (stress) or following return to LL for 12 h. Figure S1. PageMan representation of gene expression data from plants harvested at the end of the final HL photoperiod. Data were expressed as log2 fold changes of plants exposed to HL [file PCE-41-1083-s001.zip › Figure S5.pdf]
